# An atmospheric chronology for the glacial-deglacial Eastern Equatorial Pacific

**DOI:** 10.1038/s41467-018-05574-x

**Published:** 2018-08-06

**Authors:** Ning Zhao, Lloyd D. Keigwin

**Affiliations:** 1 Massachusetts Institute of Technology—Woods Hole Oceanographic Institution Joint Program in Oceanography, Woods Hole, MA USA; 2 Climate Geochemistry Department, Max Planck Institute for Chemistry, Mainz, RP Germany; 3 Present Address: Geology and Geophysics Department, Woods Hole Oceanographic Institution, Woods Hole, MA USA

## Abstract

Paleoclimate reconstructions are only as good as their chronology. In particular, different chronological assumptions for marine sediment cores can lead to different reconstructions of ocean ventilation age and atmosphere−ocean carbon exchange history. Here we build the first high-resolution chronology that is free of the dating uncertainties common in marine sediment records, based on radiocarbon dating twigs found with computed tomography scans in two cores from the Eastern Equatorial Pacific (EEP). With this accurate chronology, we show that the ventilation ages of the EEP thermocline and intermediate waters were similar to today during the Last Glacial Maximum and deglaciation, in contradiction with previous studies. Our results suggest that the glacial respired carbon pool in the EEP was not significantly older than today, and that the deglacial strengthening of the equatorial Pacific carbon source was probably driven by low-latitude processes rather than an increased subsurface supply of upwelled carbon from high-latitude oceans.

## Abstract (web summary)

Chronological assumptions in marine sediment records can result in uncertainties in paleoenvironmental reconstructions. Here, using computed tomography to identify in situ woody debris, the authors construct a robust ^14^C chronology and reassess ventilation ages in the Eastern Equatorial Pacific during the LGM.

## Introduction

Radiocarbon (^14^C) is a powerful tracer for ocean ventilation and carbon storage in the past^1–3^. However, the proxies themselves (e.g., reconstructed ^14^C age or content of seawater with reference to the atmosphere) are uncertain because they require prior knowledge of the sample age. For the most part, a marine sediment sample is dated by making assumptions about the ^14^C reservoir age of near-surface ocean or by stratigraphic alignment to an absolutely dated and typically remote record, which is based on the assumptions of the synchronicity between different climate records. In the East Equatorial Pacific (EEP), based on correlations to higher-latitude records in the Northern Hemisphere (e.g., Greenland ice cores), some studies suggest that the thermocline was much older than today during the Last Glacial Maximum (LGM) and deglaciation (up to ~2000 years)^4,5^, which is in direct contrast with other studies nearby that assume a constant reservoir age (several hundred years) of *Neogloboquadrina dutertrei*, a thermocline-dwelling foraminiferal species that is most commonly dated in the EEP^6,7^. Reservoir ages applied to planktonic foraminifera will directly influence the estimation of deep ocean ventilation age (^14^C age with reference to the contemporaneous atmosphere) based on the ^14^C age difference between benthic and planktonic foraminifera. Ventilation age of the mid-depth (~2000–3000 m) Pacific, where today exists the oldest water globally, was reported to be almost the same during the LGM as today from some cores in the Western Equatorial Pacific (WEP)^1,8^. However, glacial ventilation ages much larger than today are reported from similar depths in the EEP after large reservoir age corrections to planktonic foraminifera^4,5^. Whether the difference in ventilation age estimates is an artifact of reservoir age correction or a real geographic contrast is not clear.

The most prominent oceanic carbon source to the atmosphere today^9^, the equatorial Pacific, is shown to be even stronger during the last deglaciation^10–12^. The large ventilation ages of the EEP thermocline based on stratigraphic correlation suggests that the deglacial carbon release was caused by the subsurface transport of upwelled old carbon from high latitudes^4^. Similarly, it has been proposed that an increased transport of old intermediate water due to strengthened Southern Ocean upwelling could explain the deglacial intermediate-depth (~500–1000 m) radiocarbon depletions in the eastern tropical Pacific^13,14^. However, no indication of such old intermediate water has been found from locations in the South Pacific that are close to the proposed old water source^3,15^.

In order to reconcile these different interpretations of surface, intermediate and deep ocean carbon history, marine records from the EEP with robust age control are urgently needed. Here, we present the first high-resolution atmospheric chronology for a deep-sea sediment core based on wood ^14^C dates, and difference those dates from the ^14^C ages of planktonic and benthic foraminifera from the same layers to derive surface and subsurface ventilation conditions. The wood (twigs) is found throughout the core with the help of X-ray computed tomography (CT) scanning and provides a direct record of the ^14^C concentration of the contemporaneous atmosphere. Based on this chronology, our foraminiferal data show that the glacial and deglacial ventilation ages of the thermocline and intermediate waters in the EEP were similar to today. They provide new perspectives on the carbon history in the surface and deep equatorial Pacific and suggest that ocean ventilation reconstructions based on chronological alignment of records, especially those far apart, such as from the tropical ocean and high latitudes (e.g., ice cores), should be treated with caution.

## Results

### A wood-based chronology for a deep-sea sediment core

**Fig. 1:**
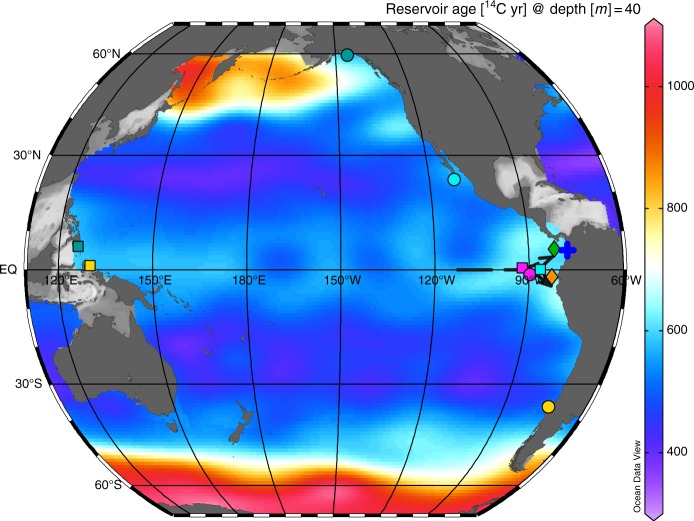
Locations of cores discussed in this paper. Blue plus: KNR176-GGC17/JPC30 from this study (5.02°N, 77.63°W, 707 m); Dots: intermediate-depth cores MV99-MC19/GC31/PC08 off Baja California (705 m, cyan)^13,32^, VM21-30 near the Galapagos (617 m, magenta)^14^, SO161-SL22 off the Chilean Margin (1000 m, yellow)^15^ and EW0408-85JC in the Gulf of Alaska (682 m, dark cyan)^38^; Squares: mid-depth cores in the EEP (TR163-23, 2730 m, magenta^4^; ODP1240, 2920 m, cyan^5^) and in the WEP (MD98-2181, 2100 m, dark cyan^1^; MD01-2386, 2800 m, yellow^8^); Diamonds: sites of an EEP oceanic Δ*p*CO_2_ record (ODP1238, orange)^10^ and of a plankton tow study in the Panama Basin (dark green)^24^. The sites are plotted with a background of the pre-bomb ^14^C reservoir age at 40 m (the median depth of the EEP thermocline^21^ and also the calcification range of *N. dutertrei* as determined from the plankton tow study^24^) from the gridded GLODAP data product^23^. EUC is shown schematically with the black dashed lines with arrow^22^. The symbol colors used in Fig. 3 follows those in this figure. Figure made with Ocean Data View^59^

Our gravity/piston-core pair (KNR176-GGC17/JPC30) is located at 707 m on the Colombian margin in the Panama Basin (Fig. 1). This site is close to the Baudó River mouth and the delta of the San Juan River (Supplementary Fig. 1). These two rivers drain one of the rainiest regions in the world, and the San Juan River has the highest water discharge on the western side of the Andes^16^. High flow speed and discharge of these short tropical mountain rivers would help fallen trees drift offshore and eventually sink in the open ocean. The narrow continental shelf along the tectonically active Pacific margin is not an effective sediment trap, especially when sea level was lower during the LGM and deglaciation, making it possible for the fallen trees and other materials of terrestrial origin to reach deep ocean.

**Fig. 2:**
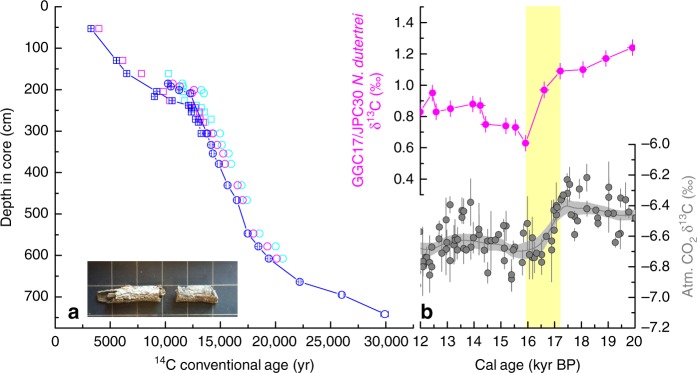
Wood-based chronology of KNR176-GGC17/JPC30. **a** ^14^C age−depth relationship (blue) of GGC17 (squares) and JPC30 (circles). Most error bars (1 s.d.) are within the symbols. Note the offset of ~30 cm between the two cores due to core-top sediment loss by the piston corer. Inserted is one example of the twigs with bark found in the cores, with 1 cm^2^ squares in the background. ^14^C ages of *N. dutertrei* and *Uvigerina* from the same layers where twigs are present are shown in magenta and cyan, respectively. **b** *N. dutertrei* δ^13^C from GGC17/JPC30 (with 1 s.d. on both axes; excluding one anomaly at ~17.4 kyr BP with unknown carbon source (Supplementary Fig. 6)) plotted with the δ^13^C of atmospheric CO_2_ reconstructed from ice cores (gray line and shaded area represent the average value and 1 s.d. envelope)^20^. Note that each δ^13^C datum is associated with a wood date. A δ^13^C decrease is apparent from ca. 17 to 16 kyr BP (as delineated by the yellow band) in both records, suggesting that good correlations to high latitudes can be achieved when there is an atmospheric teleconnection

Wood is not uncommon in deep-sea sediments^17^. We were able to find abundant twigs in GGC17/JPC30 using the density contrast between wood and sediments as revealed by CT scanning (Supplementary Fig. 2). Fallen trees are decomposed quickly on land, especially in tropical regions (on average 20% mass is estimated to be decomposed every year^18^), and wood can float only for several months before getting waterlogged and sinking^19^. In addition, most twigs found in GGC17/JPC30 still contain bark, indicating that they were fresh rather than redeposited old remains (Fig. 2a). Hence, the twigs found in the cores must have been “immediately” buried. All of the twigs have been found to lie in an approximately horizontal position in the sediments, which suggests they experienced minimal bioturbation. The radiocarbon ages of the 28 wood pieces increase with depth in the core (Fig. 2a), except for one reversal associated with a very small piece from the early Holocene where a CT image of decimeter-scale worm burrow indicates significant bioturbation (Supplementary Fig. 3; see Methods). The consistent age−depth relationship where the two cores overlap also supports the reliability of dating tree debris for establishing the chronology of deep-sea sediments (Fig. 2a). Although the stable carbon isotope composition (δ^13^C) of planktonic foraminifera could be complicated by multiple factors, further evidence for the reliability of the twig-based chronology is the synchronous variations in the δ^13^C of the planktonic foraminifera at our site and that of the atmospheric CO_2_ as determined from Antarctic ice cores^20^ (see Methods) (Fig. 2b).

### Thermocline and intermediate water ventilation in the EEP

**Fig. 3:**
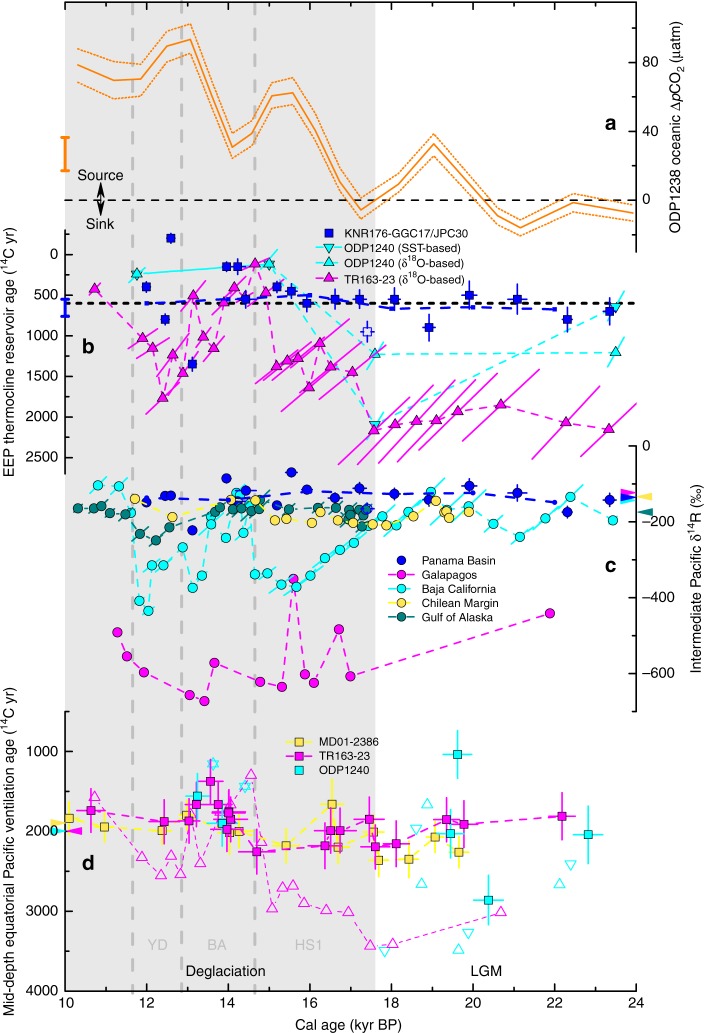
Glacial−deglacial carbon history of the surface and deep Pacific. **a** Surface oceanic Δ*p*CO_2_ from ODP1238 with envelope showing 1 s.d. uncertainties^10^. Mean mid-late Holocene (after 7 kyr BP) value from the original publication is shown with the orange bar on the left. **b** EEP thermocline ^14^C reservoir ages from KNR176-GGC17/JPC30 based on twig ^14^C (blue squares, this study), TR163-23 based on stratigraphic alignments of *G. ruber* δ^18^O (magenta triangles)^4^, and ODP1240 based on stratigraphic alignments of *G. ruber* δ^18^O (cyan triangles) and alkenone SST (cyan inverted triangles)^5^. Mean mid-late Holocene value from this study is shown with the blue bar on the left, and the pre-bomb value from GLODAP^23^ is displayed as the horizontal black dashed line. **c** Intermediate (~500–1000 m) water ^14^C disequilibria from the contemporaneous atmosphere (expressed as δ^14^R) reconstructed from KNR176-GGC17/JPC30 (blue), MV99-MC19/GC31/PC08 (cyan)^13,32^, VM21-30 (magenta)^14^, SO161-SL22 (yellow)^15,37^, and EW0408-85JC (dark cyan)^38^. Where available, the uncertainties of published records are plotted as the major axis of error ellipse. Note that for our new results in **b**, **c**, they do not vary with calendar age, and mean values at a 2000-yr step (blue dashed line) are shown to avoid interpretive artifacts imposed by variable resolution. A sample with anomalous δ^13^C and unknown carbon source at ~17.4 kyr BP (Supplementary Fig. 6) is noted with an open symbol. **d** Mid-depth (~2000–3000m) EEP ventilation ages from TR163-23 (magenta square) and ODP1240 (cyan square) reconstructed with a stable reservoir age of *N. dutertrei* from this study (see Methods). The ventilation ages based on highly variable *N. dutertrei* reservoir ages derived from stratigraphic alignments^4,5^ are displayed with open symbols (with the same colors and shapes as in **b**). ODP1240 samples with age reversals^5^ are not included. Mid-depth WEP ventilation ages from MD01-2386 are shown in yellow^8^. For **c**, **d**, modern value^23^ for each site is noted by an arrow with corresponding color on the vertical axis. All error bars in this figure represent ±1 s.d. The symbol colors used in Fig. 3 follow those in Fig. 1. YD: Younger Dryas, BA: Bølling-Allerød, HS1: Heinrich Stadial 1

*N. dutertrei* and *Uvigerina* (a benthic foraminifer) from GGC17/JPC30 were used to reconstruct ventilation history of the EEP thermocline and intermediate waters. Importantly, each datum reported in this study is associated with a twig age (Figs. 2b, 3b, c), i.e., the marine records presented here do not rely on interpolation between dated horizons. Thus, our records rely neither on assumptions about reservoir age nor on assumptions about sedimentation rate, although they could still suffer from bioturbation effects as in most marine sediment records. High sedimentation rates during most intervals in our cores (Supplementary Fig. 4) should minimize the influence of bioturbation, and the early Holocene, the interval that was the most significantly bioturbated at our site as mentioned above, is not the focus of this study. Although our site is in a region with relatively low salinity due to high precipitation and river discharge^21^, the influence of fresh water is mainly in the upper 10–15 m (Supplementary Fig. 5a). Located at ~30–50 m deep^21^, the EEP thermocline is replenished by subsurface currents from the west (e.g., the Equatorial Undercurrent (EUC); Fig. 1)^22^, so *N. dutertrei* is minimally influenced by the surface water. Planktonic foraminiferal δ^18^O data suggest the stratification caused by surface freshening has been stable since the LGM (Supplementary Fig. 5b), and the good agreement between the δ^13^C of *N. dutertrei* and *Uvigerina* at our site with those at other EEP sites (e.g., Supplementary Fig. 6) supports that our record is representative of the thermocline and intermediate-depth carbon isotope compositions in the EEP.

Six estimates of the EEP thermocline reservoir age for the LGM average 670 ± 160 yr, similar to (i) our mid-late Holocene estimates (655 ± 105 yr; *n* = 2) and (ii) modern (pre-bomb) estimates from water-column data^23^ for the calcifying depths of *N. dutertrei* in the EEP^24^ (~600 yr; Figs. 1, 3b). Although the calendar ages of our samples depend on the ^14^C calibration curve^25^, the reservoir ages reported in this study do not because they are based on coexisting twigs (atmosphere) and foraminifera (Fig. 3b). Our LGM result from the EEP is similar to that from the WEP (core MD98-2181; Fig.1), which is based on a sample also containing wood (*N. dutertrei*-wood ^14^C age difference of 780 yr and mean planktonic-wood difference of 560 yr)^1^. These suggest that the near-surface reservoir age of the glacial equatorial Pacific was not much different from today.

Radiocarbon disequilibrium between the deep ocean and the contemporaneous atmosphere is often expressed in the form of ΔΔ^14^C, the difference of Δ^14^C (the ^14^C/^12^C ratio normalized to a preindustrial atmospheric value after correction for isotopic fractionation) between the two reservoirs. However, this measure is subject to the variable atmospheric Δ^14^C. A better way to describe the disequilibrium is the ratio of ^14^C content between the two reservoirs, and we present our intermediate-depth results in the form of δ^14^R after a previous study^26^ (see Methods). For the last deglaciation, our record shows that both thermocline and intermediate waters in the EEP had radiocarbon offsets from the atmosphere that are similar to today (Fig. 3b, c). The reconstructed ventilation states are more variable between ~14–12 kyr BP (before present), although the mean values are similar to other intervals (Fig. 3b, c). This is probably due to the low sedimentation rate (10–15 cm kyr^−1^) and hence a relatively large influence of bioturbation during this interval (Supplementary Fig. 4). In particular, the negative thermocline reservoir age at ~12.6 kyr BP is unrealistic. The sample at ~17.4 kyr BP (open symbols in Fig. 3b, c) is associated with large negative δ^13^C excursions in both *N. dutertrei* and *Uvigerina*, which are not seen in other records from the EEP (Supplementary Fig. 6). The carbon source for that sample is currently not clear.

## Discussion

Our result is in stark contrast with other reconstructions of glacial thermocline ages in the EEP based on stratigraphic correlation^4,5^, which are as large as 2200 yr and even larger than the contemporaneous intermediate-depth ventilation age at our site (Fig. 3b, Supplementary Fig. 7). The thermocline depths at these EEP sites are similar, and since the EEP thermocline is closely tied to the pycnocline and chemocline^21^, it is implausible that ventilation ages were so different in similar density layers. In addition, those correlation-based reconstructions are not consistent between different proxies (e.g., alkenone SST vs. planktonic foraminiferal δ^18^O)^5^, and between different sites for the same proxy^4,5^ (Fig. 3b), which suggests that chronology determined from stratigraphic alignment is uncertain and that uncertainty propagated to the estimated ventilation age. Stratigraphic correlation suffers from uncertainties associated with, e.g., local effects on proxy records, the selection of tie points, and the phase relationship of climatic variables between distant locations (e.g., Supplementary Fig. 8). For the EEP, which may well be influenced by both hemispheres^27^, it could be very risky to synchronize records here to a higher-latitude record in one hemisphere.

If the large EEP thermocline ventilation ages based on stratigraphic correlation are an artifact of chronology, as suggested by our results, then the reported mid-depth ventilation ages^4,5^ would also be overestimates since the ^14^C age differences between benthic and planktonic foraminifera are fixed. Our thermocline reservoir age suggests that the mid-depth ventilation ages in the EEP would be very similar to those in the WEP^1,8^ (Fig. 3d). Thus, waters in the mid-depth equatorial Pacific during the LGM, where and when a large pool of respired carbon was shown to be present^28–30^, were probably not significantly older than today.

Our study also provides evidence that the previously reported deglacial anomalies of intermediate ventilation age from the eastern tropical Pacific^13,14^ do not reflect a large-scale feature. Relatively close to the EEP record near the Galapagos^14^, our record is strikingly different from it (Fig. 3c). The extremely ^14^C-depleted signal near the Galapagos probably does not represent intermediate water carbon composition^31^, otherwise it would be implausible to maintain such large gradients of radiocarbon content at similar depths in the EEP (cf. modern values in Supplementary Fig. 9). Large depletions of ^14^C during the deglaciation have been also reported from off Baja California^13,32^, at almost the same depth (705 m) as our site. These ^14^C anomalies could not be mainly due to chronological uncertainties, as the reported ^14^C age differences between benthic and planktonic foraminifera are also very large^14,32^. Nd isotope data from the Baja California site suggest that the large deglacial ^14^C age was associated with an increased influence of Equatorial Intermediate Water from the EEP and ultimately an increased contribution of Antarctic Intermediate Water from the Southern Ocean^33^. Today the influence of Southern Ocean-sourced waters (SSW) in the upper EEP (shallower than ~1000 m) is rather weak due to mixing with other mater masses^21,34^. Nd isotopes indeed show that the deep stratification in the Southern Ocean broke down during the deglaciation^35^ and the fraction of SSW in the upper EEP increased^36^. However, the fraction of SSW in the upper EEP was probably still small during the deglaciation (e.g., ~15% vs. 5% today^36^), meaning that SSW would be unrealistically old in order to explain the reported ^14^C depletions (Fig. 3c). Our record further demonstrates that the signal off Baja California could not come from the EEP.

Strengthened deep water upwelling during the deglaciation has been reported in both the Southern Ocean^2,37^ and the North Pacific^38,39^. Previous results from the Chilean Margin show relatively small intermediate-depth ventilation ages during the deglaciation^15^. However, this record has been updated with surface reservoir age constraints based on tephra layers from another core along the Chilean Margin to the south, which increased the ventilation ages earlier by ~400–900 yr^37^. Although the updated intermediate-depth ^14^C disequilibria with reference to the atmosphere were larger during the deglaciation than today and they might be overestimates due to a meridional surface reservoir age gradient along the Chilean Margin (Fig. 1), those results are still much smaller than the records off Baja California and near the Galapagos (Fig. 3c), even without considering mixing processes that could attenuate the signal of old water^40^. An intermediate-depth record from the Gulf of Alaska also showed larger ^14^C disequilibria during the deglaciation, mainly the late part (the Younger Dryas)^38^, than today (Fig. 3c). However, they are also smaller than the ^14^C anomalies in the eastern tropical Pacific. Even if the data at ~17.4 kyr BP in our record reflect a transient strong advection of isotopically light carbon from high latitudes that is too brief to be recorded in other paleo records (Supplementary Fig. 6), the ^14^C ages are only moderately larger (Fig. 3b, c). Therefore, the most parsimonious explanation for the large ^14^C anomalies is that they reflect local signals.

One possible source for those anomalies is geological carbon. The Baja California and Galapagos sites are close to mid-ocean ridges and associated hydrothermal systems (Supplementary Fig. 10). During the last deglaciation, hydrothermal activity along the East Pacific Rise (EPR) was shown to be stronger^41^, and there could be active venting systems that were closer to the two sites. The ^14^C anomalies might have been caused by hydrothermal carbon that reached the sites through local bottom water or pore water. In addition, it has been proposed that deglacial warming might have triggered CO_2_-hydrate decomposition along the EPR^31^. If those ^14^C anomalies are indeed geological carbon, its spatial influence is likely relatively limited, e.g., restricted to some sediments and water near the EPR. Our intermediate-depth record does not seem to see any influence, and the only anomalous sample in our record has a large ^13^C excursion but with only slightly enhanced ^14^C depletion (Fig. 3c, Supplementary Fig. 6), which is different from the records near Baja California and the Galapagos that have large ^14^C excursions but small ^13^C anomalies^31,42^.

As for the near-surface ocean, the large deglacial thermocline ventilation ages in the EEP estimated from TR163-23 (Fig. 3b)^4^ are probably due to chronological biases, similar to the LGM as discussed earlier. Chronological biases could contribute to the reported increase in surface water reservoir age off Baja California as well^32^, but ^14^C age difference between different planktonic foraminiferal species suggest there are other reasons^32^. It is argued that the older surface age recorded by *Globigerina ruber* was caused by seasonal upwelling of old water transported from the EEP by California Undercurrent along the coast (and an ultimate source in the Southern Ocean)^32^. Given discussions above and since those *G. ruber* ^14^C ages are even larger than the intermediate-depth ventilation ages at our site (Supplementary Fig. 7), the original explanation seems unlikely. On the other hand, geological carbon might have also played a role in the reported ^14^C anomalies of *G. ruber*, as California Undercurrent might have carried some carbon released from the hydrothermal systems at shallow depths in the Gulf of California (Supplementary Fig. 10).

Modern observations suggest that most of the carbon released from the surface equatorial Pacific into the atmosphere comes from shallow depths at low latitudes, with only a minor portion from the core of the EUC^43^. The EUC itself is a mixture of waters originating from various regions with only a small fraction from high latitudes^21,34,36^. Boron isotope data suggest that the increase in surface ocean carbon content and CO_2_ degassing in the EEP during the last deglaciation was comparable to that in the Southern Ocean (Fig. 3a)^10^. If this large increase was dominated by an increased contribution of upwelled old carbon from the Southern Ocean^2^ and/or the North Pacific^39^, then it means the fraction of water transported from high-latitude oceans was significantly larger and we should expect to see a larger ventilation age than today in the upper EEP, as suggested before^4^. However, the lack of evidence for an aging of thermocline and intermediate waters in our record instead suggests that if the equatorial Pacific CO_2_ source was strengthened during the deglaciation, it was dominated by other factor(s). For example, higher carbon leaking efficiency caused by low-latitude processes, such as thermocline shoaling and stronger upwelling in the background of a more La Niña-like mean state during the deglaciation^6,44^ could contribute to the enhanced carbon source, as El Niño-Southern Oscillation (ENSO) state significantly influences the strength of the equatorial Pacific carbon source today^43^. Weakening of upper ocean stratification in the equatorial Pacific is also a candidate^45^. Geological carbon release at intermediate depths might also contribute^31^, but it should be a minor factor as our records show that it is spatially limited.

Worthy of note is that our results do not suggest that surface water reservoir age in the equatorial Pacific did not change during the LGM and deglaciation. For example, changes of upwelling intensity in equatorial Pacific upwelling regions affect the flux of subsurface water being brought to the surface, thus contributing to the changes of surface water reservoir age as recorded by, e.g., surface-dwelling corals^11^. However, our thermocline and intermediate-depth records suggest that the glacial and deglacial age of the subsurface equatorial Pacific was relatively stable and not much older than today, so the amplitude of surface reservoir age fluctuations should be relatively limited.

Good chronology is the most fundamental component of paleo studies, and this is especially true for ^14^C records, which can be severely compromised if calendar ages are inaccurate. In addition, ^14^C variations relative to the atmosphere would change every time the calibration relationship between ^14^C age and calendar age is revised. Previous studies have shown that the radiocarbon age or concentration of the surface ocean relative to the atmosphere can be fixed using paired terrestrial plant remains and marine shells^46,47^. These records originate from coastal locations and thus are not likely to cover a long-time interval, especially for the deglaciation when the coastline moved as sea level rose. Our study suggests that if suitable locations are chosen, such as near a narrow continental shelf and/or the mouth of a river with high flow speed, core chronologies can be built for deep-sea sediments that are free of the key assumptions of previous chronologies, and robust radiocarbon history can be determined for both the surface and deep ocean. On the other hand, if tropical marine records are to be aligned with the ice cores which are located at high latitudes in one hemisphere, proxies related to the atmospheric composition (e.g., δ^13^C (refs. ^20,48,49^); Fig. 2b) might be better than those related to more local features such as temperature and precipitation (e.g., δ^18^O).

## Methods

### Acquisition of wood samples

We first observed the cross-sections of some twigs visually on the split surface of the sediment cores. We then CT-scanned the core sections at the Computerized Scanning and Imaging Facility of Woods Hole Oceanographic Institution (WHOI), and located twigs of various sizes below the split surface using the density contrast between wood and sediments. One example of the 3D CT images is displayed in Supplementary Fig. 2. Wherever possible, we dated twigs that contain bark, assuming they would have experienced shorter stay on land and no re-deposition. See Fig. 2a for a photograph of a dated twig.

### Radiocarbon analysis and ^14^C age calibration

Sample preparation and radiocarbon measurements were conducted at the National Ocean Sciences Accelerator Mass Spectrometry (NOSAMS) facility at WHOI. Wood samples were cleaned using an acid−base−acid method^50^, followed by bulk combustion. For foraminifera samples, tests were ultrasonically cleaned and then hydrolyzed with phosphoric acid. CO_2_ gas was converted to graphite using hydrogen reduction with an iron catalyzer. Graphite was then pressed into targets for Cs sputtering. Wood ^14^C ages reported from NOSAMS were calibrated to calendar ages based on IntCal13 (ref. ^25^) using Oxcal^51^. To be consistent, ^14^C dates from other studies cited in this paper were recalibrated to calendar ages, and atmospheric ^14^C ages and Δ^14^C were back calculated from given calendar ages (for ventilation age and ^14^C disequilibrium calculations in some studies) with IntCal13/Marine13 (ref. ^25^) if not done in the original publications.

### Ocean−atmosphere radiocarbon ratio

There are multiple ways to present radiocarbon concentration and the difference of radiocarbon concentrations between reservoirs. Since the radiocarbon concentration in the atmosphere is not constant through time, one appropriate way to present the radiocarbon difference between the ocean and the atmosphere is the ratio between them. In this study, we rely on the relative deviation of a reservoir from the contemporaneous atmosphere^26^:

1 $${\mathrm{\delta }}^{14}{\mathrm{R}} = \left( {\frac{{{\mathrm{Fm}}_{{\mathrm{sample}}}}}{{{\mathrm{Fm}}_{{\mathrm{atm}}}}} - 1} \right) \times 1000‰,$$

where Fm is modern fraction. Since in our case, wood radiocarbon presumably reflects atmospheric radiocarbon with high accuracy, the relative deviation is expressed as:

2 $${\mathrm{\delta }}^{14}{\mathrm{R}} = \left( {\frac{{{\mathrm{Fm}}_{{\mathrm{foram}}}}}{{{\mathrm{Fm}}_{{\mathrm{wood}}}}} - 1} \right) \times 1000‰.$$

The initial radiocarbon concentration of a sample is calculated as Δ^14^C = (Fm × e^(cal age/8267)^ − 1) × 1000, where the calendar age “cal age” is in years, so δ^14^R can also be derived from:

3 $${\mathrm {\delta }}{}^{14}{\mathrm{R}} = \left( {\frac{{{{\Delta ^{14}{\mathrm{C}}_{{\mathrm{sample}}}}}/{{1000}} + 1}}{{{{\Delta ^{14}{\mathrm{C}}_{{\mathrm{atm}}}}}/{{1000}} + 1}} - 1} \right) \times 1000‰,$$

where Δ^14^C is in ‰ and Δ^14^C_atm_ is the Δ^14^C of the atmosphere when the calcite was precipitated. Equation (3) is identical to the “epsilon value” in some studies^52^.

Equation (2) does not require knowledge of Δ^14^C_atm_ and is thus independent of radiocarbon calibration curves. For the foraminifera-wood pairs, the errors in δ^14^R appear as strictly horizontal bars in time series, as δ^14^R is unaffected by calendar age uncertainty (assuming foraminifera and wood found in the same depth horizon in the core are contemporaneous; Fig. 3c). The errors along the vertical axis are calculated from the analytical uncertainties of Fm (shown in Supplementary Table 1) with the error propagation of Eq. (2).

### Thermocline reservoir age in the EEP

Since the most commonly dated planktonic foraminifera in the EEP, *N. dutertrei*, calcifies near the thermocline (~25–50 m from an EEP plankton tow site^24^ (Fig. 1)), the thermocline ^14^C reservoir age is important for the chronologies of EEP paleo records and the ventilation age estimates of deeper waters in the EEP (once the ^14^C age difference between benthic and planktonic foraminifera is determined). By directly taking the difference between wood and *N. dutertrei* ^14^C ages, our reconstructed EEP thermocline reservoir ages are independent of sample calendar ages and of ^14^C calibration curves, i.e., the error bars related to calendar age in our study are horizontal (Fig. 3b). The error bars along the *y-*axis in Fig. 3b only include the analytical uncertainties in the ^14^C dates (for cited records, they are included in the error ellipses that are represented by the major axes as in the original publications). In order to avoid interpretive biases in our records caused by variable resolution (and also bioturbation), mean values are derived for the data (within 1 s.d. calendar age uncertainties) falling in bins of a constant duration (2ky, e.g., 11–13 and 13–15 kyr BP, given data density and climate interval boundaries; Fig. 3b, c) as suggested by a reviewer.

Our study shows that the EEP thermocline reservoir age was quite stable since the LGM (on average 590 ± 270 yr). Although there might be slight differences between the thermocline ventilation ages at our site (5.02°N, 77.63°W) and at TR163-23 (0.41°N, 92.16°W) and ODP1240 (0.02°N, 86.46°W) (Fig. 1), the mid-depth ventilation ages for TR163-23 and ODP1240 based on the EEP thermocline reservoir age derived in this study are dramatically different from previous estimates based on stratigraphic correlation^4,5^ (Fig. 3d). The ^14^C reservoir age of planktonic foraminifera used for MD01-2386 from the WEP was 560 ± 150 yr^8^, similar to the value we use for the EEP cores (590 ± 270 yr). The effect of atmospheric CO_2_ concentration on surface reservoir age^53^ is not considered here, since it is likely included in the uncertainties and it will not affect the EEP-WEP comparison in Fig. 3d, i.e., this effect has similar influences in the two regions.

### Stable carbon isotope analysis

We acquired the δ^13^C of foraminiferal tests (*N. dutertrei* and *Uvigerina*) using CO_2_ splits from the gases for ^14^C measurements. Thus, each δ^13^C datum is associated with a wood date (Supplementary Table 2). Stable isotope measurements were made on a VG Prism instrument at NOSAMS. Instrument precision is about 0.05‰.

The variation in δ^13^C of planktonic foraminifera should generally follow that of the atmospheric CO_2_ due to air−sea gas exchange (in situ or elsewhere). For *N. dutertrei* in the EEP, its δ^13^C was dominated by EUC^54^ that incorporates waters ventilated in various regions with a mean transit time of few decades^34^ (negligible given the chronological uncertainties during the deglaciation). On top of that, δ^13^C of planktonic foraminifera is also influenced by other factors, such as SST that affects the isotopic fractionation between atmospheric CO_2_ and dissolved inorganic carbon (DIC) in seawater^55^, and carbon ion concentration that impacts the fractionation when foraminifera build their shells with DIC^56^. However, although these factors can influence the variation magnitude of planktonic foraminiferal δ^13^C, they are not likely to dominate the large shift of δ^13^C between ~17–16 kyr BP (~0.4‰), which is comparable to that of the atmospheric δ^13^C (Fig. 2b), for at least two reasons. (1) Their effects are canceled by each other. For instance, the air−sea equilibrium fractionation decreases by ~0.1‰ per 1 °C increase in SST^55^, so a deglacial 3–4 °C warming for most of the surface oceans^57^ would lead to a ~0.3–0.4‰ decrease in the δ^13^C of surface ocean DIC. The lower atmospheric CO_2_ level during the LGM would cause a higher surface ocean [${\mathrm{CO}}_{3}^{2 -}$] of at least 40 μmol kg^−1^ than today, which suggests a ~0.25–0.5‰ increase of planktonic foraminiferal δ^13^C during the deglaciation, depending on species^56^. These deglacial changes of similar amplitude but opposite signs would cancel their effects to some degree. (2) Compared to previous studies from the EEP (e.g., ref. ^54^), the high sedimentation rates and individually dated δ^13^C data in this study allow us to narrow the interval of the large planktonic foraminiferal δ^13^C decrease to within about 1000 yr. We are not aware of any environmental changes besides the change of atmospheric δ^13^C that can cause such a large δ^13^C decrease. Take SST as an example; it would require an increase of ~4 °C during ~17–16 kyr BP, which is even larger than the SST increase in the Panama Basin during the whole deglacial period and that increase did not start until the later part of the Heinrich Stadial 1 (after 16 kyr BP)^7,58^.

### Bioturbation

The fact that the twigs are horizontally laid in the sediments indicates that they have not been moved much by the benthic fauna. Although it is not easy to move the horizontal twigs vertically in the sediment column due to large resistance, foraminifera shells can be much more easily moved. Thus, foraminifera samples could still be bioturbated even if our age−depth model (Fig. 2a) suggests that twigs are minimally influenced by bioturbation. There is only one reversal in the wood ^14^C ages, which stems from a small piece of wood sampled from the early Holocene segment of the core, suggesting significant bioturbation during that interval. Other evidence for significant bioturbation during the early Holocene include: (1) *Uvigerina* abundance is much lower than the intervals below and the ^14^C age difference between *Uvigerina* and wood is anomalously large (Fig. 2a; Supplementary Table 1), which suggests a net upward transport of *Uvigerina* along the sediment column. (2) A CT scanning image clearly shows a worm burrow that extends vertically over a distance of more than 30 cm (Supplementary Fig. 3). Another segment of the core where we see indications of bioturbation corresponds to the Bølling-Allerød−Younger Dryas complex (~14–12 kyr BP) when sedimentation rate was lower than during other intervals (Supplementary Fig. 4). For example, planktonic foraminifera minus wood ^14^C age is negative at 226.5 cm (~12.6 kyr BP) in GGC17, which is obviously problematic and probably reflects bioturbation influence (Fig. 3b).

### Data availability

Data generated during this study are included in the Supplementary Information file.

## Electronic supplementary material

Supplementary Information

Peer Review File
