# Supplementary material for: An atmospheric chronology for the glacial-deglacial Eastern Equatorial Pacific
Source: Nat Commun. 2018 Aug 6;9:3077. doi: 10.1038/s41467-018-05574-x (PMC6079080; doi:10.1038/s41467-018-05574-x)
Supplement: Supplementary file 1 — Supplementary Information [file 41467_2018_5574_MOESM1_ESM.pdf]

An Atmospheric Chronology for the Glacial-Deglacial Eastern Equatorial Pacific

Ning Zhao, Lloyd Keigwin

Supplementary Information

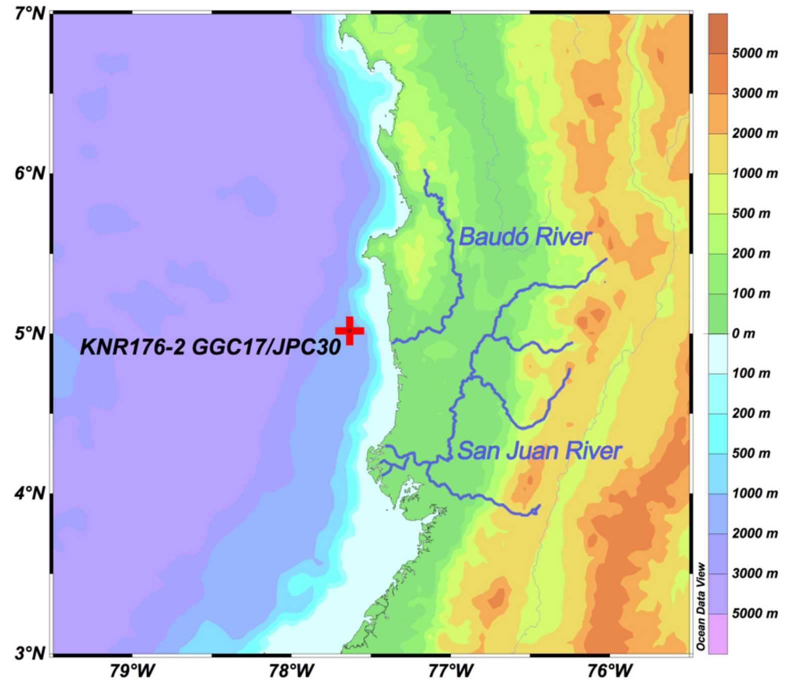

Supplementary Figure 1.

Location of KNR176-GGC17/JPC30 in an expanded map with topography. Map made with Ocean Data View[1].

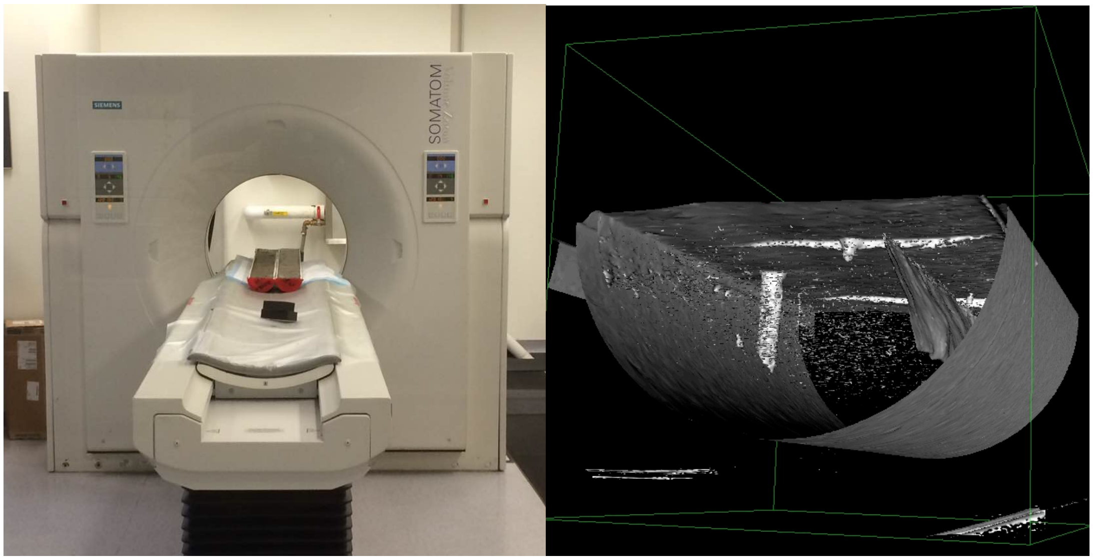

Supplementary Figure 2.

CT scanning of sediment core sections. Left: Sediment core sections being scanned by a CT machine in the Computerized Scanning and Imaging Facility at WHOI. Right: A CT scanning image revealing a twig buried in the sediments.

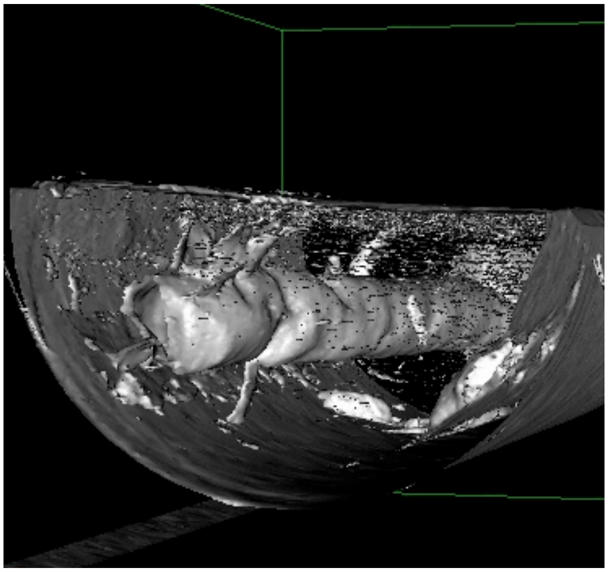

Supplementary Figure 3.

CT image showing a worm burrow. This hollow burrow extends more than 30 cm in the early Holocene sediments of GGC17.

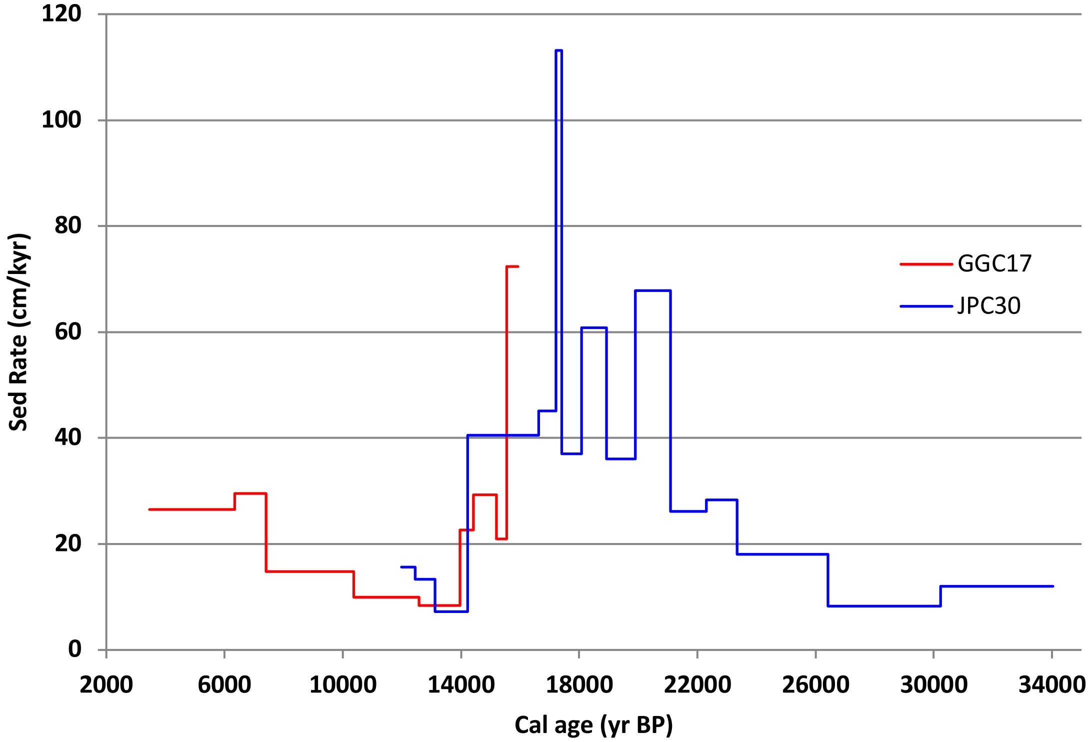

Supplementary Figure 4.
Sedimentation rate of KNR176-GGC17/JPC30.

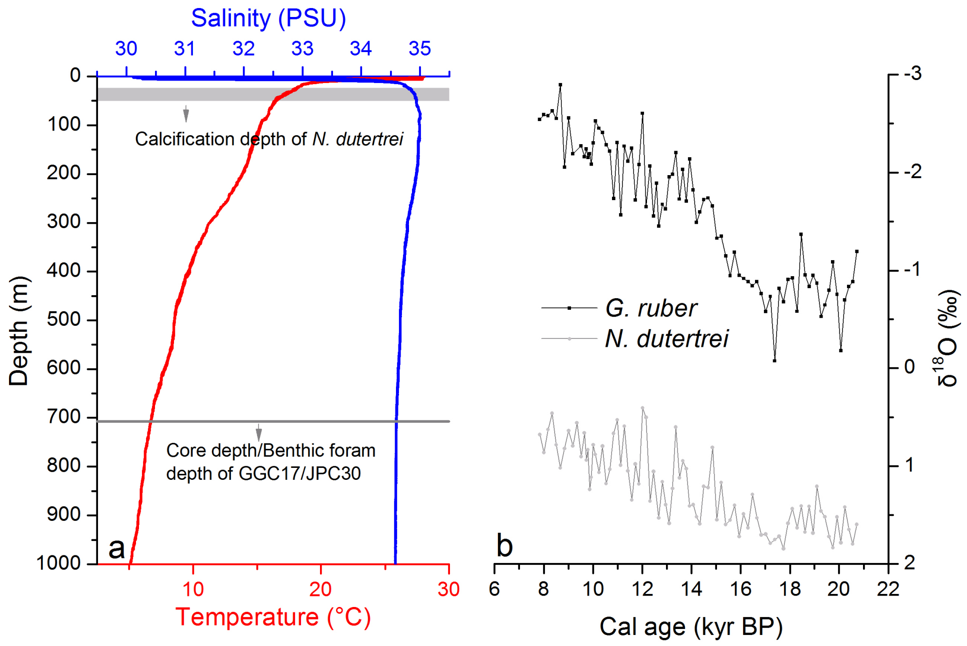

Supplementary Figure 5.

Strong surface ocean stratification near our site. (a) Temperature and salinity profiles from station KNR176-HC38 (5.00°N, 77.75°W) close to our site. According to a plankton tow study in the Panama Basin[2], *N. dutertrei* calcifies near the thermocline which is mainly influenced by subsurface currents from the west[3]. (b) *N. dutertrei* and *G. ruber* $\delta^{18}O$ from a nearby site (KNR176-JPC32, 4.67°N, 77.96°W; unpublished) suggesting strong and stable surface stratification from the LGM to the Holocene near the delta of San Juan River. The *N. dutertrei* $\delta^{18}O$ data agree well with those from other locations in the EEP (e.g., an LGM-Holocene shift from ~2‰ to ~0.8‰ at ODP1240 [4]).

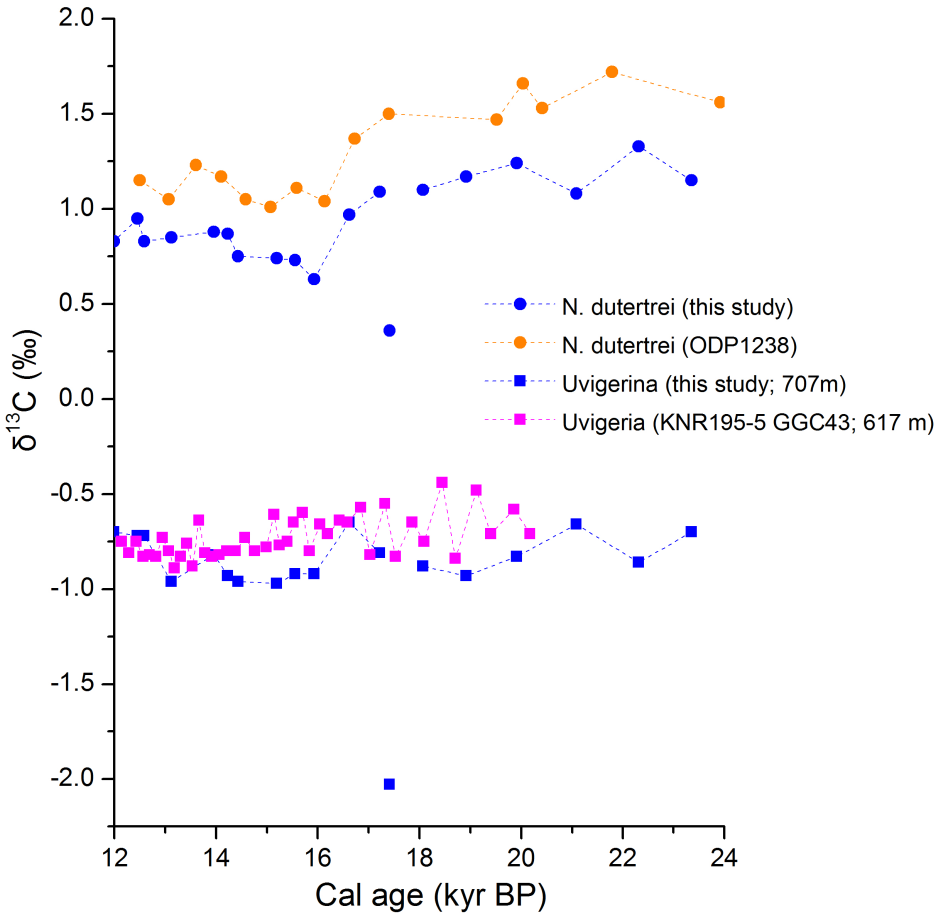

Supplementary Figure 6.

N. dutertrei (dots) and *Uvigerina* (squares) $\delta^{13}C$ from GGC17/JPC30 compared to other EEP records (*N. dutertrei* $\delta^{13}C$ from ODP1238 in the upwelling region[5] and *Uvigerina* $\delta^{13}C$ from KNR195-5 GGC43 [6] at the same site as VM21-30; Fig. 1). For the large negative excursions of $\delta^{13}C$ at ~17.4 kyr BP (isolated points), the carbon source is currently not clear.

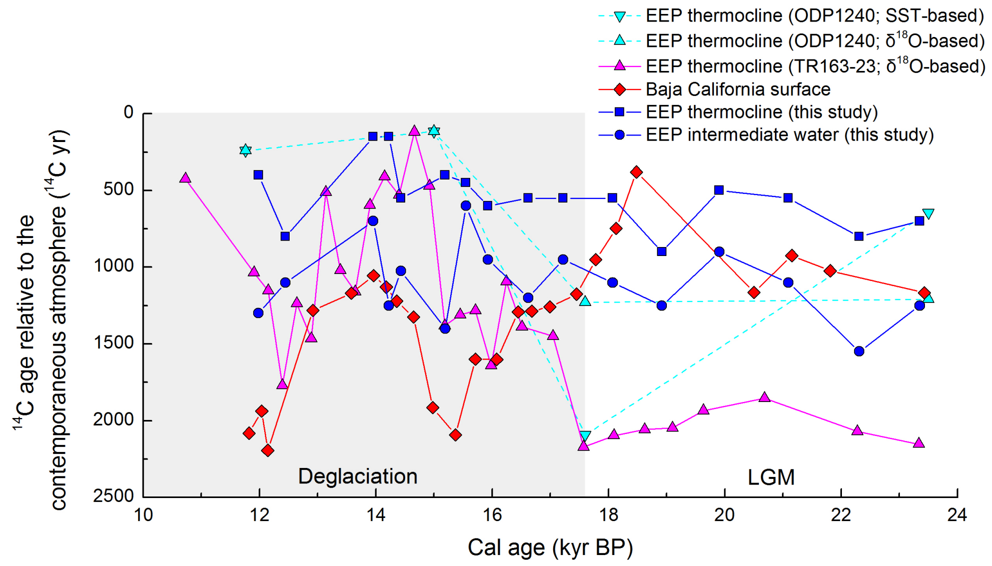

Supplementary Figure 7.

Published near-surface ^{14}C reservoir ages compared with the EEP thermocline and intermediate water ventilation ages from this study. Note that the EEP thermocline reservoir ages based on stratigraphic alignments (ODP1240 [7] and TR163-23 [8]) for most of the LGM and many deglacial samples are even larger than the intermediate-depth ventilation age in this study. The reservoir ages recorded by *G. ruber* off Baja California[9], with chronologies based on the correlation of the third factor (after R-mode factor analysis) of diffuse spectral reflectance to Greenland ice cores[10], are much larger than our thermocline and intermediate-depth ventilation ages in the EEP during the deglaciation. For clarity, the errors bars are not plotted. See Fig. 3 for a sense of the uncertainties.

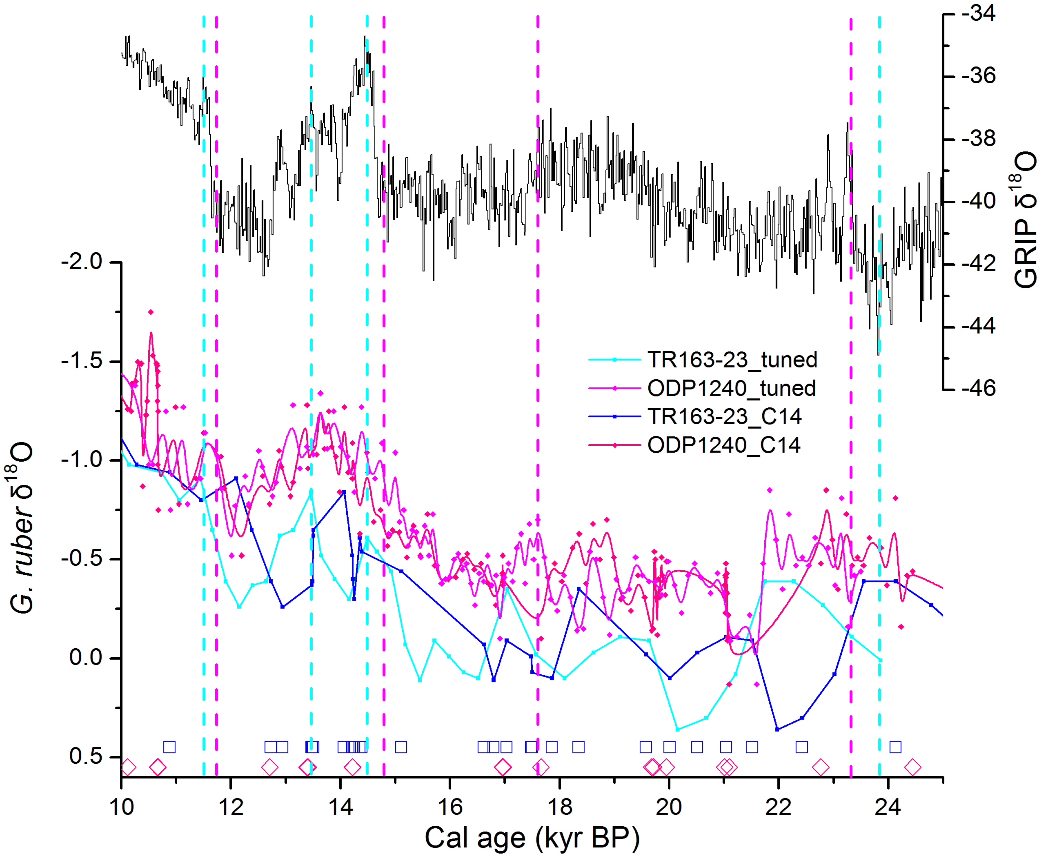

Supplementary Figure 8.

G. ruber δ^{18}O from two EEP cores plotted with the age models determined from *N. dutertrei* ^{14}C dates calibrated with a stable reservoir age (590+/-270 ^{14}C yr averaged from this study) and from *G. ruber* δ^{18}O aligned with Greenland ice core δ^{18}O (GRIP data; Ref: [11]), respectively. Vertical dashed lines delineate the tie points used, which are different between the studies (Refs: [8] for TR163-23 (cyan) and [7] for ODP1240 (magenta)). Open symbols at the bottom show the ^{14}C dates from the two cores (Refs: [8] for TR163-23 (blue) and [4,8] for ODP1240 (pink)). Note there are ^{14}C age reversals in both cores (which produces very steep slopes in the *G. ruber* δ^{18}O, e.g., ~13.5 kyr BP for TR163-23 and ~21 kyr BP for ODP1240), suggesting significant bioturbation influence. There are uncertainties associated with selections of tie points and also the synchronicities between *G. ruber* δ^{18}O in the EEP and Greenland ice core δ^{18}O. For example, *G. ruber* δ^{18}O based on calibrated ^{14}C dates shows a general decreasing trend (note the inverted axis) during ~17.5-14.5 kyr BP for both records, suggesting a feature similar to the Antarctic rather than Greenland[12]. In addition, *G. ruber* δ^{18}O between the two sites do not always agree, which might indicate local complications of the proxy.

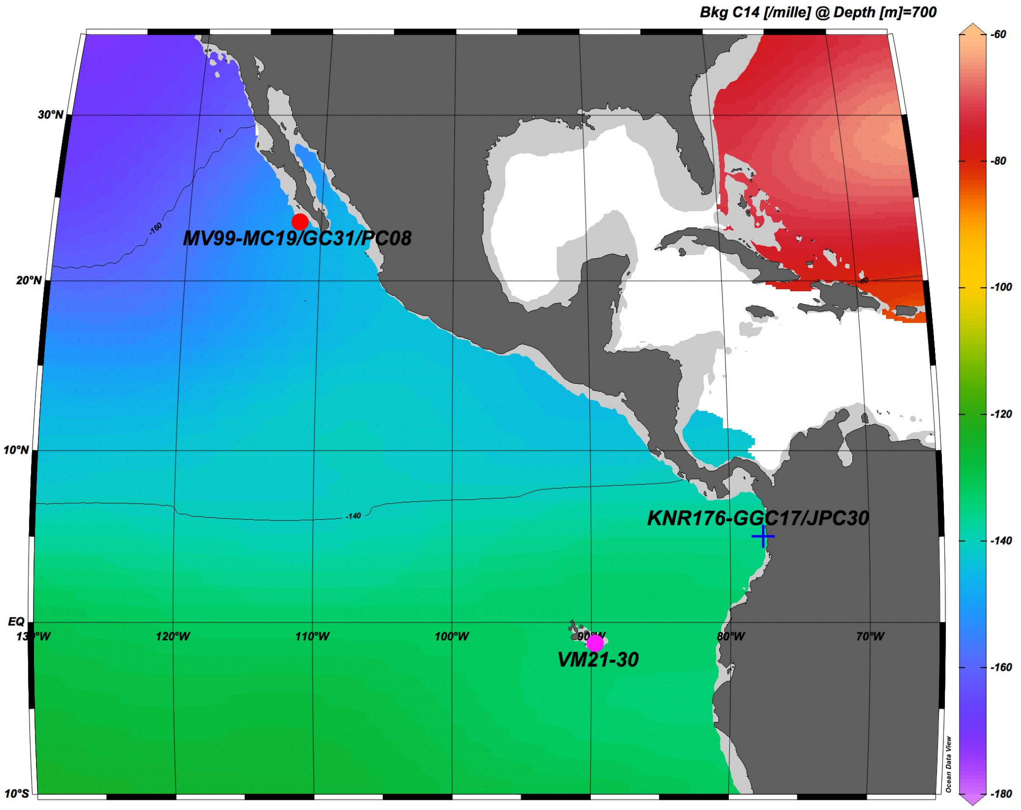

Supplementary Figure 9.

Locations of the intermediate-depth cores in the eastern tropical Pacific plotted with pre-bomb $\Delta^{14}C$ distribution at 700 m (data from gridded GLODAP product[13]). The modern $\Delta^{14}C$ differences, which are also $\delta^{14}R$ differences ($\Delta^{14}C_{atm} = 0$ for modern pre-bomb atmosphere in equation (3) of the Methods section), between these sites are no larger than 20‰. It is not plausible to maintain $\delta^{14}R$ gradients larger than 200‰ between these sites from similar depths, especially between our site and Galapagos, if all the data in Fig. 3c represent large scale features. Figure made with Ocean Data View[1].

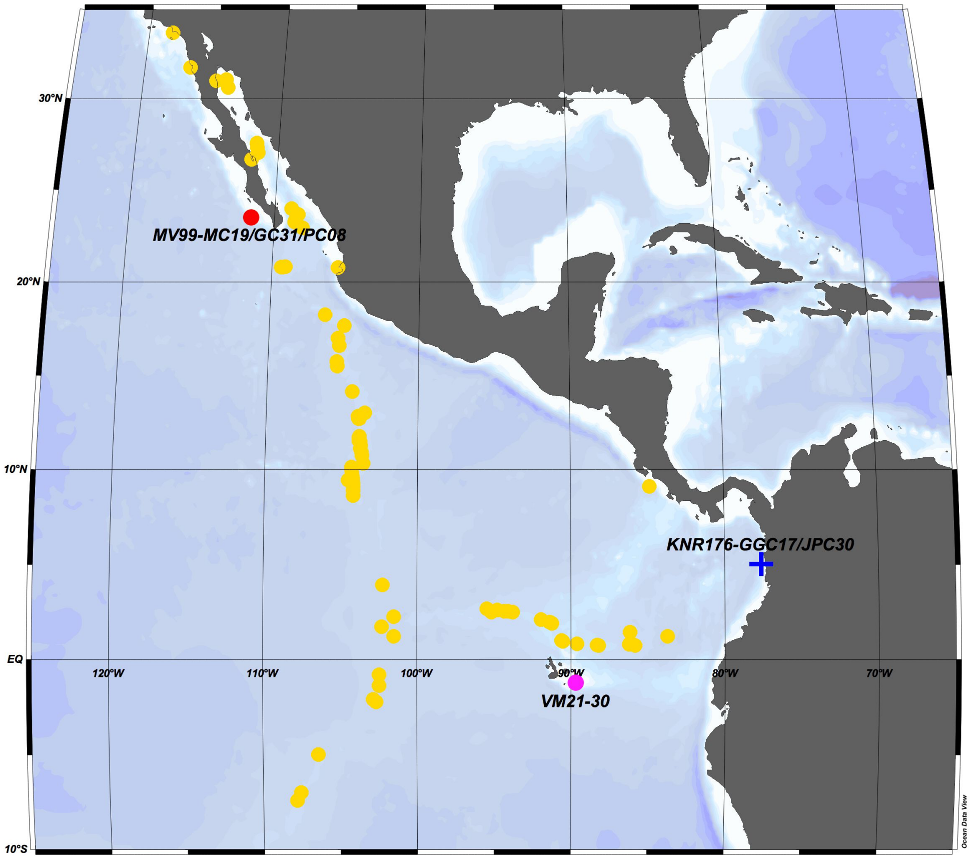

Supplementary Figure. 10.

Locations of the intermediate-depth cores with reference to active hydrothermal systems today. The observed sites of hydrothermal venting systems in the Eastern Equatorial and Northeastern Pacific are from InterRidge Vents Database Ver. 3.3 (http://vents-data.interridge.org/ventfields_list_all). Strengthened hydrothermal activities[14] and/or increased intermediate-depth temperatures[15] could lead to greater release of geological carbon near the ridges during the deglaciation. Note this map only shows the active venting sites today. There were likely more active sites during the deglaciation[14] and some could be very close to the sites of the Galapagos and Baja California records. Figure made using Ocean Data View[1].

Supplementary Table 1.

Modern fraction (Fm) and ^{14}C age of wood, planktonic and benthic foraminifera samples from KRN176-GGC17/JPC30

	Depth (cm)	Wood				*N. dutertrei*				*Uvigerina*			
		Fm	1 s.d	^{14}C age (yr)	1 s.d. (yr)	Fm	1 s.d	^{14}C age (yr)	1 s.d (yr)	Fm	1 s.d	^{14}C age (yr)	1 s.d (yr)
GGC17	53	0.6678	0.0018	3240	20	0.6098	0.0016	3970	20				
	129.5	0.5008	0.0016	5560	25	0.4654	0.0015	6140	25				
	161*	0.4454	0.0014	6500	25	0.3773	0.0014	7830	30	0.2781	0.0014	10300	40
	204.5*	0.3178	0.0018	9210	45	0.2949	0.0015	9810	40	0.2364	0.0016	11600	55
	216.5*	0.3256	0.0016	9010	40								
	226.5	0.2671	0.0015	10600	45	0.2747	0.0014	10400	40	0.2320	0.0014	11750	50
	238	0.2216	0.0010	12100	35	0.2183	0.0012	12250	45	0.2026	0.0015	12800	60
	243.5**	0.2135	0.0019	12400	70	0.2039	0.0012	12750	50	0.1924	0.0011	13250	45
	254**	0.2144	0.0011	12350	40	0.1958	0.0012	13100	50	0.1852	0.0012	13550	55
	271	0.2042	0.0012	12750	45	0.1947	0.0012	13150	50	0.1722	0.0015	14150	70
	278.5	0.1979	0.0018	13000	75	0.1873	0.0012	13450	50	0.1841	0.0011	13600	45
	306	0.1924	0.0019	13250	80	0.1782	0.0015	13850	70	0.1702	0.0011	14200	50
JPC30	185	0.2799	0.0015	10250	45	0.2661	0.0015	10650	45	0.2382	0.0014	11550	50
	192	0.2713	0.0017	10500	50	0.2457	0.0013	11300	45	0.2355	0.0015	11600	50
	201	0.2464	0.0016	11250	55	0.2083	0.0016	12600	65	0.1917	0.0015	13250	65
	209	0.2179	0.0023	12250	85	0.2136	0.0011	12400	45	0.1861	0.0015	13500	65
	306	0.1806	0.0021	13750	95	0.1687	0.0016	14300	75	0.1559	0.0014	14950	70
	333	0.1718	0.0022	14150	100	0.1604	0.0011	14700	55	0.1526	0.0016	15100	80
	354.5	0.1682	0.0023	14300	110	0.1499	0.0012	15250	65	0.1404	0.0014	15750	80
	379	0.1571	0.0021	14850	110	0.1468	0.0016	15400	85	0.1372	0.0015	15950	85
	430.5	0.1422	0.0022	15650	130	0.1278	0.0016	16550	100	0.1221	0.0014	16900	90
	466	0.1280	0.0022	16500	140	0.1204	0.0015	17000	100	0.1145	0.0015	17400	100
	546.5	0.1137	0.0022	17450	160	0.1064	0.0012	18000	90	0.0996	0.0015	18550	120
	578.5	0.1006	0.0011	18450	90	0.0911	0.0013	19250	120	0.0831	0.0012	20000	120
	608	0.0894	0.0011	19400	100	0.0818	0.0013	20100	130	0.0767	0.0012	20650	130
	663.5	0.0629	0.0008	22200	100								
	695	0.0394	0.0010	26000	210								
	741	0.0243	0.0011	29900	350								

Note:

* Early Holocene samples that are significantly influenced by bioturbation (see Methods for details)

** These two wood ^{14}C ages are the same within 1 s.d., so we combine data from the two samples.

Supplementary Table 2.

N. dutertrei and *Uvigerina* $\delta^{13}C$ of KRN176-GGC17/JPC30. Instrument precision of $\delta^{13}C$ is ~0.05‰.

	Depth (cm)	Cal age (kyr BP)	1 s.d (kyr)	*N. dutertrei* $\delta^{13}C$ (‰)	*Uvigerina* $\delta^{13}C$ (‰)
GGC17	226.5	12.59	0.06	0.83	-0.72
	238	13.96	0.08	0.88	-0.82
	243.5+254	14.44	0.18	0.75	-0.96
	271	15.19	0.08	0.74	-0.97
	278.5	15.55	0.14	0.73	-0.92
	306	15.93	0.13	0.63	-0.92
JPC30	185	11.99	0.10	0.83	-0.7
	192	12.45	0.10	0.95	-0.72
	201	13.12	0.05	0.85	-0.96
	209	14.23	0.19	0.87	-0.93
	306	16.62	0.17	0.97	-0.65
	333	17.22	0.16	1.09	-0.81
	354.5	17.41	0.16	0.36	-2.03
	379	18.07	0.14	1.1	-0.88
	430.5	18.92	0.15	1.17	-0.93
	466	19.91	0.18	1.24	-0.83
	546.5	21.09	0.23	1.08	-0.66
	578.5	22.31	0.12	1.33	-0.86
	608	23.35	0.16	1.15	-0.7

Supplementary References

1 Schlitzer, R. Ocean Data View. http://odv.awi.de (2016).

2 Fairbanks, R. G., Sverdlove, M., Free, R., Wiebe, P. H. & Bé, A. W. H. Vertical distribution and isotopic fractionation of living planktonic foraminifera from the Panama Basin. *Nature* **298**, 841-844 (1982).

3 Kessler, W. S. The circulation of the eastern tropical Pacific: A review. *Prog. Oceanogr.* **69**, 181-217 (2006).

4 Pena, L. D., Cacho, I., Ferretti, P. & Hall, M. A. El Niño–Southern Oscillation–like variability during glacial terminations and interlatitudinal teleconnections. *Paleoceanography* **23**, PA3101 (2008).

5 Martínez-Botí, M. A. *et al.* Boron isotope evidence for oceanic carbon dioxide leakage during the last deglaciation. *Nature* **518**, 219-222 (2015).

6 Bova, S. C. *et al.* Links between eastern equatorial Pacific stratification and atmospheric CO2 rise during the last deglaciation. *Paleoceanography* **30**, 1407-1424 (2015).

7 de la Fuente, M., Skinner, L., Calvo, E., Pelejero, C. & Cacho, I. Increased reservoir ages and poorly ventilated deep waters inferred in the glacial Eastern Equatorial Pacific. *Nat. Commun.* **6**, 7420 (2015).

8 Umling, N. E. & Thunell, R. C. Synchronous deglacial thermocline and deep-water ventilation in the eastern equatorial Pacific. *Nat. Commun.* **8**, 14203 (2017).

9 Lindsay, C. M., Lehman, S. J., Marchitto, T. M. & Ortiz, J. D. The surface expression of radiocarbon anomalies near Baja California during deglaciation. *Earth Planet. Sci. Lett.* **422**, 67-74 (2015).

10 Marchitto, T. M., Lehman, S. J., Ortiz, J. D., Fluckiger, J. & van Geen, A. Marine radiocarbon evidence for the mechanism of deglacial atmospheric CO_2 rise. *Science* **316**, 1456-1459 (2007).

11 Rasmussen, S. O. *et al.* A stratigraphic framework for abrupt climatic changes during the Last Glacial period based on three synchronized Greenland ice-core records: refining and extending the INTIMATE event stratigraphy. *Quat. Sci. Rev.* **106**, 14-28 (2014).

12 Koutavas, A. Temperature correlations between the eastern equatorial Pacific and Antarctica over the past 230,000 years. *Earth Planet. Sci. Lett.* **485**, 43-54 (2018).

13 Key, R. M. *et al.* A global ocean carbon climatology: Results from Global Data Analysis Project (GLODAP). *Global Biogeochem. Cycles* **18**, GB4031, doi:10.1029/2004GB002247 (2004).

14 Lund, D. C. *et al.* Enhanced East Pacific Rise hydrothermal activity during the last two glacial terminations. *Science* **351**, 478-482 (2016).

15 Stott, L. & Timmermann, A. in *Abrupt Climate Change: Mechanisms, Patterns, and Impacts* (eds Harunur Rashid, Leonid Polyak, & Ellen Mosley-Thompson) 123-138 (AGU Geophysical Monograph Series vol. 193, American Geophysical Union, 2011).
