# Supplementary material for: An atmospheric chronology for the glacial-deglacial Eastern Equatorial Pacific
Source: Nat Commun. 2018 Aug 6;9:3077. doi: 10.1038/s41467-018-05574-x (PMC6079080; doi:10.1038/s41467-018-05574-x)
Supplement: Supplementary file 2 — Peer Review File [file 41467_2018_5574_MOESM2_ESM.pdf]

Reviewers' comments:

Reviewer #1 (Remarks to the Author):

This manuscript presents a high-resolution foraminiferal 14C record of East Equatorial Pacific (EEP) thermocline and intermediate water ventilation, interpreted on an absolute chronology developed through 14C analyses on terrestrial macrofossils. This is a careful piece of work, and the use of CT-scanner to identify fresh terrestrial macrofossils is brilliant. I'm overall really impressed with both the methodology and the data.

That said, as currently written the discussion in the manuscript appears to be throwing some babies out with the bath water and fails to demonstrate that the study site is representative of open-ocean EEP conditions.

The authors conclude, based on cores KNR176-GGC17/JPC30 from a river-dominated environment on the Columbia margin, that the ventilation anomalies observed at other Eastern Pacific sites during deglaciation may be an artifact of age models developed via tuning to Greenland. While I very much share the authors' discomfort with this approach to dating Pacific records, there are a number of East Pacific records dated via the assumption of constant surface reservoir age and/or tephra correlation, as well as records developed via other proxies (e.g. εNd), that also support deglacial episodes of old intermediate-depth waters in the Pacific, likely reflecting lateral transport of an upwelled old watermass from the Southern Ocean.

Specifically, this manuscript takes aim at the conclusions of Umling and Thunell (2017) and de la Fuente et al., (2015), speculating that their findings of an increase in deglacial EEP surface ocean and intermediate/mid-depth reservoir ages may be due to age model artifacts imposed by tuning. The intermediate-depth Pacific reconstructions of Marchitto et al., 2007 (Baja California composite record MV99-MC19/GC31/PC08) and Stott et al., 2009 (Eastern Equatorial Pacific core VM21-30) are subjected to similar dismissal, and the authors of the manuscript under review speculate that the ventilation anomalies (if real) may be due to local hydrothermal input.

The authors of the planktic 14C record eventually published for the Baja core (Lindsay et al, 2015) specifically evaluated the assumption of constant surface ocean reservoir age at that site. This was in direct response to Davies-Walczak et al., 2014 raising the specter of artifacts associated with a tuned age model being responsible for much of the structure of the original Marchitto et al., 2007 intermediate water ventilation record. Lindsay et al., 2015 stated that the assumption of constant surface ocean reservoir age did not substantially change the intermediate water reconstruction, and imposed 'unlikely' sedimentation rate artifacts. This might be worth digging in to a little further, but that's what made it through review.

Basak et al., 2010 also published an εNd record from the same Baja site apparently confirming the observations of an increase in Southern Ocean sourced water at the same time as the ventilation anomalies during Heinrich Stadial 1 and perhaps in the Younger Dryas. It looks to me like their strongest evidence for a ventilation anomaly in earlier HS1 hinges on just one data point in that core, but these observations appear to be further supported by a recent compilation of Southern Ocean εNd very recently published by Basak et al. in Science ("Breakup of last glacial deep stratification in the South Pacific"). Events of similar timing in the intermediate-depth East Pacific have been observed in 14C records dated assuming constant surface reservoir age on the predominantly downwelling margin of the Gulf of Alaska (Davies-Walczak et al., 2014), a site distant from any identified vent field that I am aware of, and in 14C records from the Southern Ocean with an age model constrained by terrestrial dates on tephras (Siani et al., 2015). Note that the latter manuscript, co-authored by De Pol-Holz, posits that the lack of evidence for an increase in deglacial intermediate-water ventilation age in the published 14C record of SO161-SL122 (De Pol-Holz et al., 2010) may have been due to an incorrect assumption of constant surface ocean reservoir age in the original manuscript. Boron isotope evidence from ODP 1238 (a site featured in

this manuscript) also supports outgassing of CO_2 from the deep ocean in the EEP, presumably from the Southern Ocean, during the deglaciation.

Unfortunately the resolution of the GGC17/JPC30 data set is quite low between 12-14ka, making it difficult to say anything definitive about the younger of the two deglacial ventilation anomalies. The earlier deglaciation, 14-18ka has much better coverage and indeed appears to show local stability in both surface and intermediate depth 14C age. However, I'm not sure I'm convinced there was a good reason to discard the benthic-planktic pair showing radiocarbon depletion in the approximate window of H1 (shown in supplementary figure 8). Depletion in δ13C accompanying depletion in radiocarbon content appears to be one of the signatures of that intermediate-depth watermass in early H1 in the Pacific (e.g. Siani et al., 2015), which may (or may not) have partially vented in the EEP. Could this be actual signal?

Again, I think the methods used in the study under review are brilliant (everyone should CT scan for twigs!), and I'm all for caution when it comes to tuning climate records between hemispheres. But I also think extrapolating the results from the GGC17/JPC30 record to discount all the aforementioned (and there are more) multi-proxy observations of deglacial ventilation change in both hemispheres of the east Pacific stretching from the Southern Ocean to the Gulf of Alaska is a pretty significant overreach. This is particularly true given the site location on a fluvially-dominated margin.

One of the challenges in interpreting the record presented in this manuscript is understanding the influence of the proximity of the sample site to the mouth of the Baudó and San Juan rivers. The authors explicitly state that these river systems "drain one of the rainiest regions in the world, and the San Juan River has the highest water discharge on the western side of the Andes". I note that sedimentation rates shown in supplementary figure 4 are quite variable (although the authors really should bin this data to a constant time-step to avoid interpretive artifacts imposed by variable resolution), and appear to peak during the deglaciation. Presumably this reflects greater fluvial transport to the margin. Is it possible that local freshwater input and ensuing stratification could be influencing the ventilation structure at this site, perhaps variably in time? To me this is the big hanging question: how representative is the site of EEP upwelling environments. This could be readily evaluated, I think, by examining stable isotopic properties benthic and planktic δ18O/δ13C and comparing to one or more of the offshore locations at similar latitude. The flat structure of the d13C record in Supplementary Figure 8 really doesn't look much like the open ocean d13C record from the EEP. Are there (ideally LGM-Holocene) stable oxygen isotope records available for this core, and if so could this figure be made? Similarly, could a supplementary figure be provided of a salinity transect offshore from the site?

Hopefully these comments are useful to the authors in improving the manuscript, which overall reflects some solid science.

Reviewer #2 (Remarks to the Author):

The authors present new records of Eastern Equatorial Pacific thermocline and intermediate depth ventilation age during the LGM and deglaciation. The existence of a large respired carbon pool in the subsurface Pacific Ocean is a major question in understanding Glacial-Interglacial climate change and global climate sensitivities to CO_2. The authors use an established method to determine ventilation ages that relies on the difference in 14C ages between shallow and deep-dwelling foraminifera and that of the contemporaneous atmosphere. One difficulty with this method is that the 14C age of the atmosphere is often difficult to reconstruct in deep sea sediment cores. Researchers often have to derive a calendar age chronology using limited tie points to known age events (i.e., tephras), or patterns of climate change linked to terrestrial archives such as layer-counted ice cores or U/Th-dated speleothems. In addition, the 14C age of the ocean

includes an offset from the atmosphere, or “reservoir age”, that can be quite large and, importantly, can vary through time. The combination of poorly constrained calendar and reservoir ages in marine sediments introduces considerable uncertainty into estimates of past subsurface ventilation age. The current study site is unique in combining a narrow continental shelf with large steep tropical rivers that deliver terrestrial vegetation (wood fragments) directly to the ocean floor. The authors capitalize on this by targeting small twigs for 14C dating, providing a 14C chronology tied directly to the atmosphere and free from unknown changes in surface ocean reservoir age. The twig dates are paired with foraminiferal dates from thermocline and benthic species to calculate the ventilation age of those subsurface layers. The new results show that ventilation ages of the thermocline and intermediate waters during the LGM and deglaciation were not different from today. This disagrees with previous results from the EEP, mostly from climate-tuned calendar chronologies, that showed large reservoir age increases, and subsequent large ventilation ages, during those periods. The authors attribute these discrepancies to the previous studies – due to either incorrect assumptions regarding simultaneous climate shifts and thus inaccurate calendar chronologies, or possibly hydrothermal vent activity near those sites contributing old CO_2 to the benthic 14C ages.

Overall, this manuscript presents largely robust data sets showing little or no changes in thermoclione and intermediate water ventilations ages during the LGM and deglaciation relative to today. The potential importance and value of the manuscript relies on the quality of the chronologies. For the atmospheric 14C chronology derived from terrestrial twigs, the authors target relatively large twigs, mostly still possessing fragile bark layers, suggesting limited time spent in transport or resuspension prior to deposition and burial. One twig date appears anomalously young (and apparently the foraminiferal dates appear anomalously old, although the data are not included in Supplemental Table 1) during the early Holocene, amid evidence for local burrowing and bioturbation. Otherwise, the atmospheric 14C chronology appears consistent and of very high quality. The foraminiferal data sets also appear to be good quality, although the authors suggest that formaminifera in another segment of the sediment core were influenced by bioturbation, possibly due to low sedimentation rates during that deglacial period. However, the only evidence for bioturbation lies in anomalously high or low ventilation ages themselves. In one instance the calculated ventilation age is negative, clearly indicating post-depositional reworking of the sediments. But several other foraminferal dates simply show much larger ventilation ages than adjacent samples. As the authors note, these ventilation ages are not unrealistic, and agree somewhat with previous studies. Nevertheless, the authors dismiss the ages from discussion because they are too old, then claim that there are no old ventilation ages in the dataset. This is circular reasoning and must be corrected. Essentially, the authors need independent evidence for bioturbation if they are to invoke it as the reason for dismissing 14C ages. The authors have detailed CT scans of the cores, and quantitative measures of bioturbation should be achievable if desired. Or if the reduced sedimentation rates are sufficient cause for suspecting bioturbation, then all dates occurring within that section must be rejected. However, in the absence of such independent evidence for bioturbation influencing those samples, the authors cannot justify removing selected data points from the record, and the anomalous points must be both presented and discussed. Ultimately, I don’t believe this will significantly alter the main points of the paper. Neither thermocline nor intermediate water 14C ages show a significant shift across the end of the LGM and early deglaciation, and the great majority of points agree with modern pre-bomb values. Any anomalous points that cannot be justified for elimination would fall during the late deglaciation and early Holocene, and though a potential distraction, would not negate the findings of unchanged ventilation during the LGM relative to today.

Another, relatively minor, concern is the emphatic caution in the Abstract against linking climate chronologies to distant records. Although the caution is warranted, the authors are merely commenting on previously published interpretations of data. Even in the main text, the comment only occupies the last two sentences, and certainly does not merit inclusion in the Abstract. It seems a distraction from the major points involving the lack of ventilation changes in this region and the implications for Glacial ocean circulation. In general, the paper is clearly written and the

data interpretations are reasonable and support the stated conclusions. These new records provide valuable evidence to help resolve an important controversy and are appropriate for the readership of Nature Communications. I recommend that the paper be accepted, but pending major revision to address the above concerns.

Reviewer #3 (Remarks to the Author):

The study of Zhao and Keigwin presents new 14C data measured on twigs alongside planktic and benthic foraminifera from a sediment core in the East Equatorial Pacific over the LGM and subsequent deglaciation. The study uses the twig radiocarbon data as an atmospheric chronology, allowing for a direct comparison to be made between the foraminiferal data and the atmosphere without the need for correlating proxy records to mid- and low- latitude reference records. This is a novel approach for building an independent sediment core chronology and allows for a re-evaluation of other 14C data in the region. The main conclusion of this study is that both sub-thermocline and mid-depth ocean reservoir ages were similar to modern during the LGM and deglaciation, which is significantly different to previous studies. As a result of the novel approach and results, this paper will be of great interest to the community. The manuscript is well written and as a result I only have minor follow up questions and suggestions for improvement.

The sediment core used in the study is located on the Columbian margin close to the drainage of two major rivers. This setting is in part the reason for the abundance of wood in the core. However, is it possible that the coastal location means that the core is not representative of open ocean changes? Could this explain the discrepancy between this new work and previous studies?

It would be good to see the discussion regarding the implications for using sediment core proxy record correlated to high latitude reference records expanded in the paper. It would be interesting to see how the proxy records from cores ODP 1240 and TR163-23 compare to the Greenland and Hulu cave records when plotted on age models calculated using the thermocline reservoir age estimates from this study. Is it possible to quantify any potential leads and lags between the shifts in the proxy records from the EEP and the high latitudes or can the offsets be explained by age uncertainties in the intervals between poorly constrained tie points?

Line 70: Is there any uncertainty associated with the transport time of the twigs from tree growth location to the deep water site? Could this be significant?
Lines 90-94: This sentence is a little hard to follow. Would we expect the d13C in sub-thermocline dwelling foraminifera to track the atmosphere?
Line 120: It might be useful to have a definition of sub-thermocline, intermediate and deep water depths to ease the discussion.
Line 131: How do the proxy records look from cores ODP 1240 and TR163-23, compared to the Greenland and Hulu cave records when plotted on age models calculated using the thermocline reservoir age estimates from this study? Does it imply large leads and lags in the system? Or do the proxy records still match with the reference records within the matching error?
Line 134: mid-depth needs to be defined earlier in the paragraph (or even better earlier in the discussion).
Lines 147-148: Are there also dating uncertainties associated with these cores? Is an alternative depleted source required to explain the results? Is there evidence for this in the δ13C?
Line 345: Does the upwelling of respired δ13C not influence the signal?
Line 389: Post-depositional processes such as bioturbation?

Figure 1: It would be useful to convert 14C data into radiocarbon years as this is used throughout the paper
Figure 3: It would be useful to have a clearer distinction of the definition of intermediate versus mid-depth ages.

Reply to Reviewers

Reviewer 1

This manuscript presents a high-resolution foraminiferal 14C record of East Equatorial Pacific (EEP) thermocline and intermediate water ventilation, interpreted on an absolute chronology developed through 14C analyses on terrestrial macrofossils. This is a careful piece of work, and the use of CT-scanner to identify fresh terrestrial macrofossils is brilliant. I'm overall really impressed with both the methodology and the data.

Thank you very much for your support to our study!

That said, as currently written the discussion in the manuscript appears to be throwing some babies out with the bath water and fails to demonstrate that the study site is representative of open-ocean EEP conditions.

Please see the detailed replies below for the discussion of our results in a larger Pacific context and for our site's representativeness of open-ocean EEP conditions. Since the latter is the basis, we show our reply to the second issue first.

(moved up as it is important to clear this first) One of the challenges in interpreting the record presented in this manuscript is understanding the influence of the proximity of the sample site to the mouth of the Baudó and San Juan rivers. The authors explicitly state that these river systems "drain one of the rainiest regions in the world, and the San Juan River has the highest water discharge on the western side of the Andes". I note that sedimentation rates shown in supplementary figure 4 are quite variable (although the authors really should bin this data to a constant time-step to avoid interpretive artifacts imposed by variable resolution), and appear to peak during the deglaciation. Presumably this reflects greater fluvial transport to the margin. Is it possible that local freshwater input and ensuing stratification could be influencing the ventilation structure at this site, perhaps variably in time? To me this is the big hanging question: how representative is the site of EEP upwelling environments. This could be readily evaluated, I think, by examining stable isotopic properties benthic and planktic δ18O/δ13C and comparing to one or more of the offshore locations at similar latitude. The flat structure of the d13C record in Supplementary Figure 8 really doesn't look much like the open ocean d13C record from the EEP. Are there (ideally LGM-Holocene) stable oxygen isotope records available for this core, and if so could this figure be made? Similarly, could a supplementary figure be provided of a salinity transect offshore from the site?

In order to show our site's representativeness of the thermocline and intermediate-depth carbon isotope composition in the EEP, we have done two things as suggested. First, we now

present a salinity profile as Supplementary Fig. 5a based on a CTD cast (KNR176-HC38) very close to our site. This shows that the influence of fresh water is mainly in the upper 10-15m at our site. Although the upwelling is restrained northward of the equator in the EEP at least in part due to the surface freshening, the thermocline, which is closely tied to pycnocline and chemocline in this region, is at similar depths in the EEP (Fiedler and Talley, 2006). Located at ~30-50m deep (Fiedler and Talley, 2006), the EEP thermocline is replenished by subsurface currents from the west (Kessler, 2006). Therefore, *N. dutertrei* is minimally influenced by the surface water at our site. Located at ~700m, *Uvigerina* at our site is not subject to the influence of surface freshening.

Second, we show planktonic foram δ^{18}O from a nearby site and compare the δ^{13}C of *N. dutertrei* and *Uvigerina* at our site with those at other EEP sites (Supplementary Fig. 6) to show that the surface stratification was stable and our representativeness of EEP thermocline and intermediate-depth carbon isotope composition was valid in the LGM-Holocene transition. As mentioned in the manuscript, the δ^{13}C in our study was determined from CO_2 splits from the radiocarbon measurements. Since there was no strict temperature control during the phosphoric acid hydrolization for ^{14}C analysis, δ^{18}O of the samples were not reliable and thus not reported by NOSAMS. However, our unpublished δ^{18}O data of *G. ruber* (surface-dweller) and *N. dutertrei* (thermocline-dweller) from a nearby core (KNR176-JPC32: 4.67°N, 77.96°W) show that the surface stratification has been very strong and stable since the LGM and *N. dutertrei* at our site was not likely to be influenced much by the surface water (see Supplementary Fig. 5b). In addition, the *N. dutertrei* δ^{18}O from KNR176-JPC32 is very close to that in other EEP cores (e.g., there is an LGM-Holocene shift from ~2‰ to ~0.8‰ at ODP1240 (Pena et al., 2008); cf. Supplementary Fig. 5b).

The plot for *N. dutertrei* and *Uvigerina* δ^{13}C at our site was originally focused on the large anomaly at ~17.4kyr BP, so the vertical axis was not expanded enough to show the structures. Now it has been elongated so that we can see more details rather than two flat lines, although the benthic record is indeed quite flat as discussed below. The *N. dutertrei* δ^{13}C record between our site and ODP1238 in the upwelling zone south of the Equator (the core used in the boron isotope and ΔpCO_2 study cited in Fig. 3a of our paper) agree well. The slightly more negative δ^{13}C at our site might reflect a deeper calcification depth of *N. dutertrei* at our site with reference to the chemocline, which is associated with a large δ^{13}C gradient (Fairbanks et al., 1982). As for benthic foraminifera, there are not many δ^{13}C records available from similar depths to our site (~700m) in the EEP. It was mentioned that there is no large δ^{13}C anomaly for the *Uvigerina* in the Galapagos core VM21-30 (617m) (Stott and Timmermann, 2011), but the data were not reported. However, we found *Uvigerina* δ^{13}C data from a core at the same location, KNR195-5 GGC43 (Bova et al., 2015), which agrees well with our record and also shows a relatively flat trend (Supplementary Fig. 6). δ^{13}C of *Uvigerina* and *C. wuellerstorfi* from the mid-depth (2000-3000m) EEP show an increasing trend from the LGM to the Holocene, although the shift is not very large (~0.5‰) (Umling and Thunell, 2018). These might suggest that waters at ~600-700m in the EEP was not influenced as much by a changing respired carbon reservoir since the LGM.

The paper has been revised to reflect the main points mentioned above (Lines 114-123 and Supplementary Figs. 5&6).

In addition, as suggested, we have now binned our data to a constant time-step (2000 yr as shown in Fig.3 given the resolution of our data) to reduce interpretive artifacts imposed by variable resolution.

The authors conclude, based on cores KNR176-GGC17/JPC30 from a river-dominated environment on the Columbia margin, that the ventilation anomalies observed at other Eastern Pacific sites during deglaciation may be an artifact of age models developed via tuning to Greenland. While I very much share the authors' discomfort with this approach to dating Pacific records, there are a number of East Pacific records dated via the assumption of constant surface reservoir age and/or tephra correlation, as well as records developed via other proxies (e.g. εNd), that also support deglacial episodes of old intermediate-depth waters in the Pacific, likely reflecting lateral transport of an upwelled old watermass from the Southern Ocean.

Specifically, this manuscript takes aim at the conclusions of Umling and Thunell (2017) and de la Fuente et al., (2015), speculating that their findings of an increase in deglacial EEP surface ocean and intermediate/mid-depth reservoir ages may be due to age model artifacts imposed by tuning. The intermediate-depth Pacific reconstructions of Marchitto et al., 2007 (Baja California composite record MV99-MC19/GC31/PC08) and Stott et al., 2009 (Eastern Equatorial Pacific core VM21-30) are subjected to similar dismissal, and the authors of the manuscript under review speculate that the ventilation anomalies (if real) may be due to local hydrothermal input.

The authors of the planktic 14C record eventually published for the Baja core (Lindsay et al, 2015) specifically evaluated the assumption of constant surface ocean reservoir age at that site. This was in direct response to Davies-Walczak et al., 2014 raising the specter of artifacts associated with a tuned age model being responsible for much of the structure of the original Marchitto et al., 2007 intermediate water ventilation record. Lindsay et al., 2015 stated that the assumption of constant surface ocean reservoir age did not substantially change the intermediate water reconstruction, and imposed 'unlikely' sedimentation rate artifacts. This might be worth digging in to a little further, but that's what made it through review.

Basak et al., 2010 also published an εNd record from the same Baja site apparently confirming the observations of an increase in Southern Ocean sourced water at the same time as the ventilation anomalies during Heinrich Stadial 1 and perhaps in the Younger Dryas. It looks to me like their strongest evidence for a ventilation anomaly in earlier HS1 hinges on just one data point in that core, but these observations appear to be further supported by a recent compilation of Southern Ocean εNd very recently published by Basak et al. in Science ("Breakup of last glacial deep stratification in the South Pacific"). Events of similar timing in the intermediate-depth East Pacific have been observed in 14C records dated assuming constant surface reservoir age on the predominantly downwelling margin of the Gulf of Alaska (Davies-Walczak et al., 2014), a site distant from any identified vent field that I am aware of, and in 14C records from the Southern Ocean with an age model constrained by terrestrial dates on tephras (Siani et al., 2015). Note that the latter manuscript, co-authored by De Pol-Holz, posits that the lack of evidence for an increase in deglacial intermediate-water ventilation age in the published 14C record of SO161-SL122 (De Pol-Holz et al., 2010) may have been due to an incorrect assumption of constant surface ocean reservoir age in the original manuscript. Boron isotope evidence from ODP 1238 (a site featured in this manuscript) also supports outgassing of CO_2 from the deep ocean in the EEP, presumably from the Southern Ocean, during the deglaciation.

Thanks for the nice summary of our study and other records in the Pacific.

Since our site is in the Eastern Equatorial Pacific (EEP), we only focused on discussions about records in the tropical Pacific in the previous version. We did not mean to negate

records of intermediate-depth ventilation age in the higher-latitude Pacific. We apologize that the title was likely misleading as we were trying to shorten it and only kept “East Pacific”. “Eastern Equatorial Pacific” is now used in the title to be more accurate. In the previous version, we only briefly referred to some records at higher latitudes, so it is great that Reviewer 1 gives us an opportunity here to discuss our results more in the context of records in the broader Pacific. The intermediate-depth records from higher latitudes have been included in Fig. 3c and also in the map (Fig. 1).

Siani et al. (2013; we think the reviewer meant 2013 based on our knowledge and searching) and Davies-Walczak et al. (2014) are now cited with other studies from high latitudes to show the evidence that old water upwelled during the deglaciation in the Southern Ocean (Burke and Robinson, 2012; Siani et al., 2013) and the North Pacific (Davies-Walczak et al., 2014; Rae et al., 2014). We now point out that the Chilean Margin record by De Pol-Holz et al. (2010) has been updated with surface reservoir age constraints based on tephra layers from Siani et al. (2013), which increased the ventilation ages earlier by ~400-900 yr. Since Siani et al.’s site is located at 1536m and is bathed today by the upper Pacific deep Water near its boundary with Antarctic Intermediate Water (Siani et al., 2013), we use the record from De Pol-Holz et al. (2010) (1000m) but with the updated reservoir age constraints by (Siani et al., 2013) in the compilation of Pacific intermediate-depth (~500-1000m) ventilation state (Fig. 3c). We state that the record by Davies-Walczak et al. (2014) and the updated record from De Pol-Holz et al. (2010) had larger ^{14}C offsets from the atmosphere than today. On the other hand, these offsets are still much smaller than the records from off Baja California (Marchitto et al., 2007) and the Galapagos (Stott et al., 2009), even without considering mixing processes that could attenuate the signal of old water as it flows from high to low latitudes (Hain et al., 2011).

We have added that Nd isotope data from the Baja California site suggest the large deglacial ^{14}C age was associated with an increased influence of Equatorial Intermediate Water from the EEP and ultimately an increased contribution of Antarctic Intermediate Water from the Southern Ocean (Basak et al., 2010). We also added that Nd isotopes show the deep stratification in the Southern Ocean broke down during the deglaciation (Basak et al., 2018) and the fraction of SSW in the upper EEP increased (Pena et al., 2013). On the other hand, the influence of Southern Ocean-sourced waters (SSW) in the upper EEP (shallower than ~1000m) is rather weak today due to mixing with other mater masses (Fiedler and Talley, 2006; Goodman et al., 2005), and the fraction of SSW in the upper EEP was probably still small during the deglaciation (e.g., as estimated from Nd isotope data: ~15% vs 5% today (Pena et al., 2013)). Therefore, the Southern Ocean-sourced intermediate waters would need to be unrealistically old in order to explain the reported ^{14}C depletions near Baja California and Galapagos after mixing with other water masses.

We now make it clear that the ^{14}C anomalies near Baja California and the Galapagos could not be mainly due to chronological uncertainties (e.g., surface ocean reservoir age), as the reported ^{14}C age differences between benthic and planktonic foraminifera are also very large (Lindsay et al., 2015; Stott et al., 2009). Therefore, based on the several points above, the most parsimonious explanation for the large ^{14}C anomalies is that they reflect local signals, and one possible source is hydrothermal influence as discussed in our manuscript.

We clarify further that our records suggest if the equatorial Pacific CO_2 source was strengthened during the deglaciation, it cannot be mainly due to an increased subsurface supply of upwelled old carbon from high-latitude oceans. As we wrote in the previous version, modern observations suggest that most of the carbon released from the equatorial

Pacific comes from shallow depths at low latitudes, with only a minor portion from the core of the EUC (Feely et al., 1999), and the EUC itself is a mixture of waters originating from various regions with only a small fraction from high latitudes (Fiedler and Talley, 2006; Goodman et al., 2005; Pena et al., 2013). The boron isotope record from ODP 1238 suggests that the increase in surface ocean carbon content and CO_2 degassing in the EEP was comparable to that in the Southern Ocean during the deglaciation (Martínez-Botí et al., 2015). Thus, if this large increase was dominated by an increased contribution of upwelled old carbon from the Southern Ocean and/or the North Pacific, then it means the fraction of water transported from high-latitude oceans was significantly larger in the upper EEP and we should expect to see a larger EEP ventilation age than today as suggested by previous studies (Umling and Thunell, 2017), which is in contrary to we observe in the EEP. Alternative explanations for the larger deglacial carbon outgassing include, e.g., higher carbon leaking efficiency caused by low-latitude processes, such as thermocline shoaling and stronger upwelling in the background of a more La Niña-like mean state during the deglaciation (Clement et al., 1999; Pena et al., 2008), and a weakening of upper ocean stratification in the equatorial Pacific (Bova et al., 2015).

The points above are now reflected in Lines 175-214 and 243-262 of our manuscript.

Unfortunately the resolution of the GGC17/JPC30 data set is quite low between 12-14ka, making it difficult to say anything definitive about the younger of the two deglacial ventilation anomalies. The earlier deglaciation, 14-18ka has much better coverage and indeed appears to show local stability in both surface and intermediate depth 14C age. However, I'm not sure I'm convinced there was a good reason to discard the benthic-planktic pair showing radiocarbon depletion in the approximate window of H1 (shown in supplementary figure 8). Depletion in δ13C accompanying depletion in radiocarbon content appears to be one of the signatures of that intermediate-depth watermass in early H1 in the Pacific (e.g. Siani et al., 2015), which may (or may not) have partially vented in the EEP. Could this be actual signal?

As now stated in Lines 142-144, the reconstructed ventilation states are more variable between ~14-12 kyr BP, probably due to bioturbation influence (e.g., the *N. dutertrei*-wood ^{14}C age difference at ~12.6 kyr BP is negative, which is apparently unrealistic and was excluded but now added back in Fig. 3b as suggested by Reviewer 2). However, the mean values at a constant time step of the thermocline reservoir age and the intermediate-depth ^{14}C offset from the atmosphere are similar to other intervals, supporting our argument that the ventilation state of the upper EEP was stable through the deglaciation (Fig. 3b&c).

As for the sample around 17.4 kyr BP, its large negative δ^{13}C excursions in both *N. dutertrei* and *Uvigerina* are not seen in other records from the EEP (Supplementary Fig. 6). Siani et al. (2013) did not show benthic δ^{13}C data directly, but based on their *G. bulloides* δ^{13}C (the lowest deglacial values around -1‰) and the negative *G. bulloides* - *C. wuellerstorfi* δ^{13}C difference, their deglacial benthic δ^{13}C is heavier than -1‰, and hence quite heavier than the benthic value we have around 17.4 kyr BP (-2.03‰). Although there was probably species difference between benthic δ^{13}C, Umling and Thunell (2018) shows *Uvigerina* δ^{13}C is only ~0.5‰ lighter than *C. wuellerstorfi* from an EEP site. The intermediate-depth δ^{13}C we observe is probably lighter than most if not all intermediate-depth Pacific records and is not likely to be a signal of large-scale water mass features. In the Results section, we write that the carbon source for this sample is currently not clear. However, in the discussion section, we now talk about what it means if the signal is real. First, if the data at ~17.4 kyr BP in our record reflect a transient strong advection of isotopically light carbon from high latitudes that

is too brief to be recorded in other paleo records, the $\delta^{14}R$ (^{14}C ratio of the intermediate-depth ocean to the atmospheric) are only moderately lower (empty symbols in Fig. 3c) and still much smaller than the deglacial depletions from Baja California and Galapagos, questioning their ^{14}C data. Second, if the records near Baja California and the Galapagos reflect hydrothermal influence, then that influence was spatially limited and did not reach our site because the most likely candidate in our record, the sample around 17.4ka BP, has a large $\delta^{13}C$ excursion but with only slightly enhanced ^{14}C depletion, which is different from the records near Baja California and the Galapagos that have large ^{14}C excursions but small ^{13}C anomalies (Lindsay et al., 2016; Stott and Timmermann, 2011). These are now included in Lines 210-213 and 224-228.

Again, I think the methods used in the study under review are brilliant (everyone should CT scan for twigs!), and I'm all for caution when it comes to tuning climate records between hemispheres. But I also think extrapolating the results from the GGC17/JPC30 record to discount all the aforementioned (and there are more) multi-proxy observations of deglacial ventilation change in both hemispheres of the east Pacific stretching from the Southern Ocean to the Gulf of Alaska is a pretty significant overreach. This is particularly true given the site location on a fluvially-dominated margin.

Thanks for your positive comment.

As we write above, we did not mean to discount all the records in the broader Pacific, and we now discuss the intermediate-depth records at higher latitudes in more detail. The representativeness of our site of the upper EEP carbon isotope evolution has also been demonstrated.

Hopefully these comments are useful to the authors in improving the manuscript, which overall reflects some solid science.

Thanks for your good suggestions and comments on our study!

Reviewer 2

The authors present new records of Eastern Equatorial Pacific thermocline and intermediate depth ventilation age during the LGM and deglaciation. The existence of a large respired carbon pool in the subsurface Pacific Ocean is a major question in understanding Glacial-Interglacial climate change and global climate sensitivities to CO2. The authors use an established method to determine ventilation ages that relies on the difference in 14C ages between shallow and deep-dwelling foraminifera and that of the contemporaneous atmosphere. One difficulty with this method is that the 14C age of the atmosphere is often difficult to reconstruct in deep sea sediment cores. Researchers often have to derive a calendar age chronology using limited tie points to known age events (i.e., tephras), or patterns of climate change linked to terrestrial archives such as layer-counted ice cores or U/Th-dated speleothems. In addition, the 14C age of the ocean includes an offset from the atmosphere, or "reservoir age", that can be quite large and, importantly, can vary through time. The combination of poorly constrained calendar and reservoir ages in marine sediments introduces considerable uncertainty into estimates of past subsurface ventilation age. The current study site is unique in combining a narrow continental shelf with large steep tropical rivers that

deliver terrestrial vegetation (wood fragments) directly to the ocean floor. The authors capitalize on this by targeting small twigs for 14C dating, providing a 14C chronology tied directly to the atmosphere and free from unknown changes in surface ocean reservoir age. The twig dates are paired with foraminiferal dates from thermocline and benthic species to calculate the ventilation age of those subsurface layers. The new results show that ventilation ages of the thermocline and intermediate waters during the LGM and deglaciation were not different from today. This disagrees with previous results from the EEP, mostly from climate-tuned calendar chronologies, that showed large reservoir age increases, and subsequent large ventilation ages, during those periods. The authors attribute these discrepancies to the previous studies – due to either incorrect assumptions regarding simultaneous climate shifts and thus inaccurate calendar chronologies, or possibly hydrothermal vent activity near those sites contributing old CO2 to the benthic 14C ages.

Thanks for the very nice summary of our study!

Overall, this manuscript presents largely robust data sets showing little or no changes in thermoclione and intermediate water ventilations ages during the LGM and deglaciation relative to today. The potential importance and value of the manuscript relies on the quality of the chronologies. For the atmospheric 14C chronology derived from terrestrial twigs, the authors target relatively large twigs, mostly still possessing fragile bark layers, suggesting limited time spent in transport or resuspension prior to deposition and burial. One twig date appears anomalously young (and apparently the foraminiferal dates appear anomalously old, although the data are not included in Supplemental Table 1) during the early Holocene, amid evidence for local burrowing and bioturbation. Otherwise, the atmospheric 14C chronology appears consistent and of very high quality. The foraminiferal data sets also appear to be good quality, although the authors suggest that formaminifera in another segment of the sediment core were influenced by bioturbation, possibly due to low sedimentation rates during that deglacial period. However, the only evidence for bioturbation lies in anomalously high or low ventilation ages themselves. In one instance the calculated ventilation age is negative, clearly indicating post-depositional reworking of the sediments. But several other foraminferal dates simply show much larger ventilation ages than adjacent samples. As the authors note, these ventilation ages are not unrealistic, and agree somewhat with previous studies. Nevertheless, the authors dismiss the ages from discussion because they are too old, then claim that there are no old ventilation ages in the dataset. This is circular reasoning and must be corrected. Essentially, the authors need independent evidence for bioturbation if they are to invoke it as the reason for dismissing 14C ages. The authors have detailed CT scans of the cores, and quantitative measures of bioturbation should be achievable if desired. Or if the reduced sedimentation rates are sufficient cause for suspecting bioturbation, then all dates occurring within that section must be rejected. However, in the absence of such independent evidence for bioturbation influencing those samples, the authors cannot justify removing selected data points from the record, and the anomalous points must be both presented and discussed. Ultimately, I don't believe this will significantly alter the main points of the paper. Neither thermocline nor intermediate water 14C ages show a significant shift across the end of the LGM and early deglaciation, and the great majority of points agree with modern pre-bomb values. Any anomalous points that cannot be justified for elimination would fall during the late deglaciation and early Holocene, and though a potential distraction, would not negate the findings of unchanged ventilation during the LGM relative to today.

Thanks for this good point!

During our lab analysis, we dated twigs before preparing foraminiferal samples. For the only twig date that is an outlier in the age-depth model, we did not date the foraminifera in that layer after we got the twig age, thus the foraminiferal dates for that sample are empty in Supp. Table 1. For other early Holocene samples, we get anomalous *Uvigerina*-twig ^{14}C age differences that are consistent with significant bioturbation in this interval as suggested by the CT image of burrow. These are now made clear in Supp. Table 1 and the Methods section. Furthermore, we have added back the ^{14}C dates that were excluded before in Fig. 3b&c. Although the variability of data is larger during ~14-12 ka BP, the mean values at a constant time-step (to remove interpretive artifacts imposed by variable resolution as suggested by Reviewer 1; Fig. 3b&c) are very similar with other intervals (Lines 142-144). Therefore, as you mentioned, the inclusion of these data does not alter the findings we report. To be consistent, we have also included a sample (at ~20kyr BP) for ODP1240 that was excluded due to its relatively young ^{14}C age (de la Fuente et al., 2015) (Fig. 3d).

As for CT scans, right now we only have one clear evidence (the hollow burrow) for bioturbation. We appreciate the idea of deriving quantitative measures of bioturbation using CT scans, but currently we do not have a clear plan how to do that. This probably deserves further investigations in the future.

Another, relatively minor, concern is the emphatic caution in the Abstract against linking climate chronologies to distant records. Although the caution is warranted, the authors are merely commenting on previously published interpretations of data. Even in the main text, the comment only occupies the last two sentences, and certainly does not merit inclusion in the Abstract. It seems a distraction from the major points involving the lack of ventilation changes in this region and the implications for Glacial ocean circulation. In general, the paper is clearly written and the data interpretations are reasonable and support the stated conclusions. These new records provide valuable evidence to help resolve an important controversy and are appropriate for the readership of Nature Communications. I recommend that the paper be accepted, but pending major revision to address the above concerns.

As suggested, the sentence of emphatic caution has been taken out from the abstract. On the other hand, stimulated by Reviewer 3, we now have a more detailed discussion about the uncertainties associated with chronologies based on stratigraphic alignments (Lines 157-166 and Supplementary Fig. 8).

Thanks for your support to our paper!

Reviewer 3

The study of Zhao and Keigwin presents new 14C data measured on twigs alongside planktic and benthic foraminifera from a sediment core in the East Equatorial Pacific over the LGM and subsequent deglaciation. The study uses the twig radiocarbon data as an atmospheric chronology, allowing for a direct comparison to be made between the foraminiferal data and the atmosphere without the need for correlating proxy records to mid- and low- latitude reference records. This is a novel approach for building an independent sediment core chronology and allows for a re-evaluation of other 14C data in the region. The main conclusion of this study is that both sub-thermocline and mid-depth ocean reservoir ages were similar to modern during the LGM and deglaciation, which is significantly different to previous studies. As a result of the novel approach and results, this paper will be of great

interest to the community. The manuscript is well written and as a result I only have minor follow up questions and suggestions for improvement.

Thank you very much for the support to our study!

The sediment core used in the study is located on the Columbian margin close to the drainage of two major rivers. This setting is in part the reason for the abundance of wood in the core. However, is it possible that the coastal location means that the core is not representative of open ocean changes? Could this explain the discrepancy between this new work and previous studies?

We have demonstrated our site's representativeness of the open ocean changes in the paper now. Please see our response to Reviewer 1 (on the first and second pages of this reply) for details.

It would be good to see the discussion regarding the implications for using sediment core proxy record correlated to high latitude reference records expanded in the paper. It would be interesting to see how the proxy records from cores ODP 1240 and TR163-23 compare to the Greenland and Hulu cave records when plotted on age models calculated using the thermocline reservoir age estimates from this study. Is it possible to quantify any potential leads and lags between the shifts in the proxy records from the EEP and the high latitudes or can the offsets be explained by age uncertainties in the intervals between poorly constrained tie points?

We now show the *G. ruber* $\delta^{18}O$ from ODP1240 and TR163-23 based on age models calculated using the thermocline reservoir age estimates from this study, and compare them with those based on the original age models and the Greenland ice core $\delta^{18}O$ (see Supplementary Fig. 8). There are uncertainties associated with selections of tie points (different tie points for the two records and some tie points are poorly constrained; Supplementary Fig. 8) and also the synchronicity between *G. ruber* $\delta^{18}O$ in the EEP and Greenland ice core $\delta^{18}O$. For example, the *G. ruber* $\delta^{18}O$ based on calibrated ^{14}C dates shows a general decreasing trend (note the inverted axis) during ~17.5-14.5 kyr BP for both records, suggesting a feature similar to the Antarctic rather than Greenland (Koutavas, 2018). In addition, *G. ruber* $\delta^{18}O$ between the two sites do not always agree, which might indicate local complications of the proxy. Therefore, it is hard and might not be feasible to quantify any potential leads and lags between the shifts in the EEP proxy records and the high-latitude records in one hemisphere. We thank the editor for this comment, which stimulates us to show more details about the uncertainties associated correlation-based chronologies (in Lines 157-166 and Supplementary Fig. 8).

Line 70: Is there any uncertainty associated with the transport time of the twigs from tree growth location to the deep water site? Could this be significant?

There is uncertainty associated with the transport time of the twigs, but this uncertainty is small (probably on the order of years) and will not be significant on the timescale we are working on and given the uncertainties of radiocarbon dating (several decades to one century, see Supp. Table 1). There are several points that support this argument: (1) The flow speed and water discharge of the short mountainous rivers in the study region is high; (2) wood can

float only for several months before getting waterlogged and sinking (Häggblom, 1982); (3) most twigs found in our cores still contain bark, indicating that they had a relatively short journey before burial and they were fresh rather than redeposited old remains. (4) Fallen trees are decomposed quickly on land, especially in tropical regions (on average 20% mass is estimated to be decomposed every year (Chambers et al., 2000)). If a twig stays on land for a decade, most of mass will be lost and we would not expect to see bark. These points are shown on Lines 87-92.

Lines 90-94: This sentence is a little hard to follow. Would we expect the d13C in sub-thermocline dwelling foraminifera to track the atmosphere?

The sub-thermocline water in the EEP is replenished by subsurface currents from the west (e.g., the Equatorial Undercurrent (EUC)) (Kessler, 2006). EUC incorporates waters ventilated in various regions but with a mean transit time of few decades (Goodman et al., 2005). This is negligible given the chronological uncertainties during the deglaciation (more than a century). So we expect the $\delta^{13}C$ of *N. dutertrei* to track the atmosphere. This is made clear in the ^{13}C part of the Methods section (Lines 375-378), which is referred to after the sentence (now on Lines 99-103).

Line 120: It might be useful to have a definition of sub-thermocline, intermediate and deep water depths to ease the discussion.

The depth of intermediate water (500-1000m) and mid-depth water (2000-3000m) discussed in this study is now defined early in the introduction. We do not define the depth of thermocline, but the thermocline depth (which is also roughly the calcification depth of *N. dutertrei* (Fairbanks et al., 1982)) in the EEP as defined in previous studies is also made clear in the paper now (~30-50m (Fiedler and Talley, 2006)) in Line 116.

Line 131: How do the proxy records look from cores ODP 1240 and TR163-23, compared to the Greenland and Hulu cave records when plotted on age models calculated using the thermocline reservoir age estimates from this study? Does it imply large leads and lags in the system? Or do the proxy records still match with the reference records within the matching error?

Please see our reply to a comment earlier (the third comment from Reviewer 3).

Line 134: mid-depth needs to be defined earlier in the paragraph (or even better earlier in the discussion).

Done.

Lines 147-148: Are there also dating uncertainties associated with these cores? Is an alternative depleted source required to explain the results? Is there evidence for this in the $\delta13C$?

Yes, there are also dating uncertainties with these cores as their chronologies are also based on stratigraphic alignments. However, these intermediate-depth ^{14}C anomalies could not be mainly due to chronological uncertainties, as the reported ^{14}C age differences between benthic and planktonic foraminifera are also very large (Lindsay et al., 2015; Stott et al., 2009). Now this is made clear in Lines 183-185. As we show in the manuscript (Lines 175-214), those anomalies cannot come from high latitudes and do not represent a large scale feature, so the most parsimonious explanation for the large ^{14}C anomalies is that they reflect local signals. We now make it clear that geological/hydrothermal carbon is only one possibility (Line 215). As discussed in Stott and Timmermann (2011), the anomalies of $\delta^{13}C$ for geological/hydrothermal carbon is not significant as they could be recycled carbonate and DIC.

Line 345: Does the upwelling of respired δ13C not influence the signal?

As we discuss in the manuscript, changes of upwelling intensity in equatorial Pacific upwelling regions affect the flux of subsurface water being brought to the surface, thus could contribute to the changes of surface water reservoir age as recorded by, e.g., surface-dwelling corals. However, our thermocline and intermediate-depth records suggest that the glacial and deglacial age of the EEP subsurface water was relatively stable and not much older than today. Similarly, upwelling of respired $\delta^{13}C$ will probably influence the surface water $\delta^{13}C$, but the subsurface $\delta^{13}C$ (e.g., recorded by *N. dutertrei*) would not be influenced much by upwelling as it is bathed all the time by the water that upwells.

Line 389: Post-depositional processes such as bioturbation?

Yes, we have now clearly state bioturbation in the sentence (see Line 421).

Figure 1: It would be useful to convert 14C data into radiocarbon years as this is used throughout the paper

Done.

Figure 3: It would be useful to have a clearer distinction of the definition of intermediate versus mid-depth ages.

Definitions of the depths have been added in the caption.

References

Basak, C., Fröllje, H., Lamy, F., Gersonde, R., Benz, V., Anderson, R. F., Molina-Kescher, M., and Pahnke, K., 2018, Breakup of last glacial deep stratification in the South Pacific: Science, v. 359, no. 6378, p. 900-904.

Basak, C., Martin, E., Horikawa, K., and Marchitto, T., 2010, Southern Ocean source of 14 C-depleted carbon in the North Pacific Ocean during the last deglaciation: Nature Geoscience, v. 3, no. 11, p. 770-773.

Bova, S. C., Herbert, T., Rosenthal, Y., Kalansky, J., Altabet, M., Chazen, C., Mojarro, A., and Zech, J., 2015, Links between eastern equatorial Pacific stratification and atmospheric CO2 rise during the last deglaciation: Paleoceanography, v. 30, no. 11, p. 1407-1424.

Burke, A., and Robinson, L. F., 2012, The Southern Ocean's role in carbon exchange during the last deglaciation: Science, v. 335, p. 557-561.

Chambers, J. Q., Higuchi, N., Schimel, J. P., Ferreira, L. V., and Melack, J. M., 2000, Decomposition and carbon cycling of dead trees in tropical forests of the central Amazon: Oecologia, v. 122, no. 3, p. 380-388.

Clement, A. C., Seager, R., and Cane, M. A., 1999, Orbital controls on the el nino/southern Oscillation and the tropical climate: Paleoceanography, v. 14, p. 441-456.

Davies-Walczak, M., Mix, A. C., Stoner, J. S., Southon, J. R., Cheseby, M., and Xuan, C., 2014, Late Glacial to Holocene radiocarbon constraints on North Pacific Intermediate Water ventilation and deglacial atmospheric CO_2 sources: Earth and Planetary Science Letters, v. 397, p. 57-66.

de la Fuente, M., Skinner, L., Calvo, E., Pelejero, C., and Cacho, I., 2015, Increased reservoir ages and poorly ventilated deep waters inferred in the glacial Eastern Equatorial Pacific: Nature Communications, v. 6, p. 7420.

De Pol-Holz, R., Keigwin, L., Southon, J., Hebbeln, D., and Mohtadi, M., 2010, No signature of abyssal carbon in intermediate waters off Chile during deglaciation: Nature Geoscience, v. 3, no. 3, p. 192-195.

Fairbanks, R. G., Sverdlove, M., Free, R., Wiebe, P. H., and Bé, A. W. H., 1982, Vertical distribution and isotopic fractionation of living planktonic foraminifera from the Panama Basin: Nature, v. 298, p. 841-844.

Feely, R. A., Wanninkhof, R., Takahashi, T., and Tans, P., 1999, Influence of El Nino on the equatorial Pacific contribution to atmospheric CO2 accumulation: Nature, v. 398, no. 6728, p. 597-601.

Fiedler, P. C., and Talley, L. D., 2006, Hydrography of the eastern tropical Pacific: A review: Progress in Oceanography, v. 69, no. 2, p. 143-180.

Goodman, P. J., Hazeleger, W., de Vries, P., and Cane, M., 2005, Pathways into the Pacific Equatorial Undercurrent: A trajectory analysis: Journal of physical oceanography, v. 35, no. 11, p. 2134-2151.

Häggblom, A., 1982, Driftwood in Svalbard as an indicator of sea ice conditions: Geografiska Annaler. Series A. Physical Geography, v. 64, p. 81-94.

Hain, M. P., Sigman, D. M., and Haug, G. H., 2011, Shortcomings of the isolated abyssal reservoir model for deglacial radiocarbon changes in the mid-depth Indo-Pacific Ocean: Geophysical Research Letters, v. 38, no. 4, p. L04604.

Kessler, W. S., 2006, The circulation of the eastern tropical Pacific: A review: Progress in Oceanography, v. 69, no. 2, p. 181-217.

Koutavas, A., 2018, Temperature correlations between the eastern equatorial Pacific and Antarctica over the past 230,000 years: Earth and Planetary Science Letters, v. 485, p. 43-54.

Lindsay, C. M., Lehman, S. J., Marchitto, T. M., Carriquiry, J. D., and Ortiz, J. D., 2016, New constraints on deglacial marine radiocarbon anomalies from a depth transect near Baja California: Paleoceanography, v. 31, no. 8, p. 1103-1116.
Lindsay, C. M., Lehman, S. J., Marchitto, T. M., and Ortiz, J. D., 2015, The surface expression of radiocarbon anomalies near Baja California during deglaciation: Earth and Planetary Science Letters, v. 422, p. 67-74.
Marchitto, T. M., Lehman, S. J., Ortiz, J. D., Fluckiger, J., and van Geen, A., 2007, Marine radiocarbon evidence for the mechanism of deglacial atmospheric CO_2 rise: Science, v. 316, p. 1456-1459.
Martínez-Botí, M. A., Marino, G., Foster, G. L., Ziveri, P., Henehan, M. J., Rae, J. W. B., Mortyn, P. G., and Vance, D., 2015, Boron isotope evidence for oceanic carbon dioxide leakage during the last deglaciation: Nature, v. 518, no. 7538, p. 219-222.
Pena, L. D., Cacho, I., Ferretti, P., and Hall, M. A., 2008, El Niño–Southern Oscillation–like variability during glacial terminations and interlatitudinal teleconnections: Paleoceanography, v. 23, no. 3, p. PA3101.
Pena, L. D., Goldstein, S. L., Hemming, S. R., Jones, K. M., Calvo, E., Pelejero, C., and Cacho, I., 2013, Rapid changes in meridional advection of Southern Ocean intermediate waters to the tropical Pacific during the last 30 kyr: Earth and Planetary Science Letters, v. 368, p. 20-32.
Rae, J. W. B., Sarnthein, M., Foster, G. L., Ridgwell, A., Grootes, P. M., and Elliott, T., 2014, Deep water formation in the North Pacific and deglacial CO_2 rise: Paleoceanography, v. 29, no. 6, p. 645-667.
Siani, G., Michel, E., De Pol-Holz, R., DeVries, T., Lamy, F., Carel, M., Isguder, G., Dewilde, F., and Lourantou, A., 2013, Carbon isotope records reveal precise timing of enhanced Southern Ocean upwelling during the last deglaciation: Nature communications, v. 4, p. 2758.
Stott, L., Southon, J., Timmermann, A., and Koutavas, A., 2009, Radiocarbon age anomaly at intermediate water depth in the Pacific Ocean during the last deglaciation: Paleoceanography, v. 24, no. 2, p. PA2223.
Stott, L., and Timmermann, A., 2011, Hypothesized link between glacial/interglacial atmospheric CO_2 cycles and storage/release of CO_2-rich fluids from deep-sea sediments, *in* Rashid, H., Polyak, L., and Mosley-Thompson, E., eds., Abrupt Climate Change: Mechanisms, Patterns, and Impacts: Washington, DC, AGU Geophysical Monograph Series vol. 193, American Geophysical Union, p. 123-138.
Umling, N. E., and Thunell, R. C., 2017, Synchronous deglacial thermocline and deep-water ventilation in the eastern equatorial Pacific: Nature communications, v. 8, p. 14203.
Umling, N. E., and Thunell, R. C., 2018, Mid-depth respired carbon storage and oxygenation of the eastern equatorial Pacific over the last 25,000 years: Quaternary Science Reviews, v. 189, p. 43-56.

REVIEWERS' COMMENTS:

Reviewer #1 (Remarks to the Author):

I feel the authors' revisions reflect a thoughtful response to my initial comments/suggestions. The interpretations of the EEP in the manuscript are as a result more balanced and the broader context of deglacial ventilation changes in the Pacific is now well represented. I still have some questions about the potential influence of stratification on the thermocline record, but it's the authors' prerogative to present a reasonable interpretation of their data, as they have, and the supplement now includes adequate information for the reader to draw their own conclusions.

The manuscript could still use a careful proof read. For example, in the revised caption for Figure 3, the deglacial portion of the Gulf of Alaska record shown will be from the jumbo piston core (or JC) as opposed to the trigger core (or TC). In line 613 values ARE, as opposed to IS shown. In that same line, it is unclear which data in panels b and c are binned to 2kyr resolution (presumably only the new data from this manuscript), and how that binning is displayed (presumably the dark blue dashed lines).

On that note, while interesting to see the effect vis a vis flattening the ventilation record, my suggestion of binning to constant resolution to avoid interpretive artifacts was more for the sed rate data shown in Supplement Figure 4. Specifically, that high sed rate interval ~17ka is probably an artifact of resolution.

These suggestions are all really minor and, while I hope they will be of some use to the authors as they finalize the manuscript, at this point I'm happy to recommend heading to publication.

Reviewer #2 (Remarks to the Author):

This is a revised version of a submission I previously reviewed. The authors have made substantial revisions to address concerns brought by reviewers, adding text and figures to clarify the scope of their interpretations and treating individual data points more objectively. The authors have adequately addressed all of the concerns in my previous review. In particular, apparently anomalous 14C dates are included in Fig 3 and discussed in some detail in the text. Since data points are no longer being excluded on the basis of potential bioturbation, there is no need for quantitative bioturbation measures to justify the exclusion. As noted, including these data points does not influence the interpretations and conclusions of the paper, and is a more transparent treatment of the data.

The discussion of possible explanations for discrepancies between this and previous studies is greatly improved, outlining potential mechanisms and citing evidence to support or refute each. The clarification of low vs high-latitude sites in the discussion is important and has been strengthened. The expanded discussion of stratigraphic alignments between long distance records is good. Showing the differences in chronologies based on twig 14C versus aligning surface foram d18O to Greenland ice cores is a strong addition to the supplemental figures. Overall, the manuscript is significantly more clear and objectively presented than previously, and I recommend it be accepted for publication in its present form.

Reviewer #3 (Remarks to the Author):

The authors have addressed all of my comments and I am happy with the changes made to the manuscript. I look forward to seeing this paper published.

One minor comment:
Figure 3(d) caption-state ‘reservoir age’ correction on N.dutertrei 14C from this study

Reply to the Reviewers

Thank you all for the good comments and suggestions!

Quoted comments from the reviewers are in black and our responses are in blue.

Reviewer 1

I feel the authors' revisions reflect a thoughtful response to my initial comments/suggestions. The interpretations of the EEP in the manuscript are as a result more balanced and the broader context of deglacial ventilation changes in the Pacific is now well represented. I still have some questions about the potential influence of stratification on the thermocline record, but it's the authors' prerogative to present a reasonable interpretation of their data, as they have, and the supplement now includes adequate information for the reader to draw their own conclusions.

Thanks for your comments!

The manuscript could still use a careful proof read. For example, in the revised caption for Figure 3, the deglacial portion of the Gulf of Alaska record shown will be from the jumbo piston core (or JC) as opposed to the trigger core (or TC). In line 613 values ARE, as opposed to IS shown. In that same line, it is unclear which data in panels b and c are binned to 2kyr resolution (presumably only the new data from this manuscript), and how that binning is displayed (presumably the dark blue dashed lines).

On that note, while interesting to see the effect vis a vis flattening the ventilation record, my suggestion of binning to constant resolution to avoid interpretive artifacts was more for the sed rate data shown in Supplement Figure 4. Specifically, that high sed rate interval ~17ka is probably an artifact of resolution.

Thank you for the very careful reading, and we have made changes in the caption of Figure 3 as suggested (see Lines 614-616). We have also checked through the manuscript carefully.

Thanks for the clarification on the binning average.

These suggestions are all really minor and, while I hope they will be of some use to the authors as they finalize the manuscript, at this point I'm happy to recommend heading to publication.

Thank you!

Reviewer 2

This is a revised version of a submission I previously reviewed. The authors have made substantial revisions to address concerns brought by reviewers, adding text and figures to clarify the scope of their interpretations and treating individual data points more objectively. The authors have adequately addressed all of the concerns in my previous review. In particular, apparently anomalous 14C dates are included in Fig 3 and discussed in some detail in the text. Since data points are no longer being excluded on the basis of potential

bioturbation, there is no need for quantitative bioturbation measures to justify the exclusion. As noted, including these data points does not influence the interpretations and conclusions of the paper, and is a more transparent treatment of the data.

The discussion of possible explanations for discrepancies between this and previous studies is greatly improved, outlining potential mechanisms and citing evidence to support or refute each. The clarification of low vs high-latitude sites in the discussion is important and has been strengthened. The expanded discussion of stratigraphic alignments between long distance records is good. Showing the differences in chronologies based on twig 14C versus aligning surface foram d18O to Greenland ice cores is a strong addition to the supplemental figures. Overall, the manuscript is significantly more clear and objectively presented than previously, and I recommend it be accepted for publication in its present form.

Thank you very much for the nice summary and your support to our paper!

Reviewer 3

The authors have addressed all of my comments and I am happy with the changes made to the manuscript. I look forward to seeing this paper published.

Thank you!

One minor comment:
Figure 3(d) caption-state ‘reservoir age’ correction on N. dutertrei 14C from this study

We have added “reservoir age” in Figure 3(d) caption.
